# *De novo* Mutations (DNMs) in Autism Spectrum Disorder (ASD): Pathway and Network Analysis

**DOI:** 10.3389/fgene.2018.00406

**Published:** 2018-09-21

**Authors:** Aitana Alonso-Gonzalez, Cristina Rodriguez-Fontenla, Angel Carracedo

**Affiliations:** ^1^Grupo de Medicina Xenómica, Fundación Instituto de Investigación Sanitaria de Santiago de Compostela, Center for Research in Molecular Medicine and Chronic Diseases, Universidade de Santiago de Compostela, Santiago, Spain; ^2^Grupo de Medicina Xenómica, CIBERER, Centre for Research in Molecular Medicine and Chronic Diseases, Universidade de Santiago de Compostela, Santiago, Spain

**Keywords:** Autism Spectrum Disorder, genetics, post-zygotic mutations, neurodevelopmental disorders, *de novo* mutations, gene networks, pathway analysis, whole exome sequencing

## Abstract

Autism Spectrum Disorder (ASD) is a neurodevelopmental disorder (NDD) defined by impairments in social communication and social interactions, accompanied by repetitive behavior and restricted interests. ASD is characterized by its clinical and etiological heterogeneity, which makes it difficult to elucidate the neurobiological mechanisms underlying its pathogenesis. Recently, *de novo* mutations (DNMs) have been recognized as strong source of genetic causality. Here, we review different aspects of the DNMs associated with ASD, including their functional annotation and classification. In addition, we also focus on the most recent advances in this area, such as the detection of PZMs (*post-zygotic mutations*), and we outline the main bioinformatics tools commonly employed to study these. Some of these approaches available allow DNMs to be analyzed in the context of gene networks and pathways, helping to shed light on the biological processes underlying ASD. To end this review, a brief insight into the future perspectives for genetic studies into ASD will be provided.

## Introduction

Autism Spectrum Disorder (ASD) includes a range of NDDs that are characterized by deficits in social communication and interactions, as well as by repetitive behaviors and restrictive interests, with onset in early development (American Psychiatric Association, 2013). The estimated prevalence of ASD in the general population stands at approximately 1%, with males being about three times more likely than females to be affected (Fombonne, 2009; Loomes et al., 2017).

Twin and family studies have demonstrated a genetic contribution to ASD etiology. Indeed, early reports showed a concordance in ASD diagnosis in monozygotic (MZ, 70–90%) and DZ twins (10%), which indicates a heritability of about 90% (Steffeneburg, 1989; Bailey et al., 1995). A recent analysis more precisely estimated heritability to be 83%, which is slightly lower than that reported in the earlier twin studies (Sandin et al., 2017). Moreover, the risk of ASD increases for a child when he has an older affected sibling and as such, the overall risk of recurrence in siblings has been estimated to be around 6.9–18% depending on the study design. This range is also influenced by whether half or full siblings are considered (Ozonoff et al., 2011; Gronborg et al., 2013; Risch et al., 2014).

A substantial fraction of this heritability can be explained by SNPs. The contribution of these common variants to ASD etiology stands at around 50% when it is additively considered (Gaugler et al., 2014). However, early GWAS failed to detect strong signals, in part due to the need for larger samples (Weiss et al., 2009; Anney et al., 2010; Ma et al., 2010). However, subsequent large-scale GWAS identified 12 novel ASD loci, some of them identified as plausible common risk variants in earlier studies (Autism Spectrum Disorders Working Group of The Psychiatric Genomics Consortium, 2017). Moreover, the latest GWAS meta-analysis conducted by the PGC not only represented an incredible effort to increase sample size up to tens of thousands of cases and controls but also, it developed a well-defined quality control and imputation pipeline. For the first time, the results of this ASD GWAS meta-analysis led to the identification of 93 significant genome-wide markers, of which 53 were replicated in independent cohorts (Grove et al., 2017).

Despite the evidence of a significant role for common variants in ASD risk, rare genetic variation (*MAF*<1%) confers higher individual risk (**Table 1**). Rare variation can be found as small insertions and deletions (indels), CNVs or SNVs. Moreover, these can be inherited from a paternal and/or maternal origin or they may appear *de novo* in the affected subject (De Rubeis et al., 2014). Such DNMs, are mutations identified in the proband that are not found in the genomes of the biological parents. The importance of DNMs in ASD genetics is strongly related to the role of natural selection and allele frequency. Therefore, rare risk alleles tend to be eliminated by purifying selection while common ones show signs of positive selection (Polimanti and Gelernter, 2017). These facts mean that DNMs are most likely to have a strong effect and thus, the discovery of DNMs allows ASD risk genes to be identified. Indeed, exons expressed in the brain that are subject to purifying selection were enriched for DNMs in ASD (Uddin et al., 2014).

**Table 1:** Genetic architecture of ASD.

% Liability due to different classes of mutations		% Of different classes of mutations harbored by ASD probands	
Common variation	49.4%		
*De novo* variation	3%	*De novo* CNVs	4–7%
		*De novo* SNVs	7%
Rare inherited variation	3%	Rare variants AR	3%
		X-linked variants	2%
Total	55%	Total	16–19%


*The liability in ASD according to the different classes of mutation and the different types of mutations harbored by ASD individuals. Data taken from Gaugler et al. (2014).*

The different types of genetic variants, combined with their distinct pattern of inheritance or their *de novo* origin, define the potential genetic risk for ASD. For example, carrying a *de novo* SNV and a specific non-sense mutation in the coding sequence confers around five times more individual risk than carrying a transmitted CNV (Stein et al., 2013). Moreover, children with severe ASD symptoms along with ID are thought to carry more harmful DNMs (Robinson et al., 2014). Hence, there is now considerable interest in identifying novel DNMs associated with ASD.

## DNMs in ASD Genetics

### Identification of DNMs

Trio genetic association studies (parents and affected proband) have been used since 2007 to study DNMs and to find mutations in the proband that were not present in either parent. By performing such studies on large cohorts of patients and controls, and by analyzing the characteristics of the DNMs identified, it is possible to characterize previously unrecognized ASD genes, the main goal of such studies. In the first studies to detect CNVs using high-resolution microarrays, *de novo* CNVs were more frequent in cases than controls (Marshall et al., 2008; Pinto et al., 2010; Sebat et al., 2010; Levy et al., 2011; Sanders et al., 2011) and also more frequent in simplex rather than multiplex families (Marshall et al., 2008; Sebat et al., 2010).

However, the large size of CNVs presents a problem when attempting to detect ASD candidate genes. Indeed, genes disrupted by CNVs may contribute to a moderate risk of ASD, whereas SNVs are more likely to directly indicate genes associated with a high susceptibility for ASD (Sanders et al., 2015). Accordingly, large scale parallel sequencing and specifically, WES has been employed widely to unravel the genetic architecture of ASD (Betancur, 2011; Buxbaum et al., 2013; Sener et al., 2016). Indeed, the vast majority of DNM studies have employed this technology, in conjunction with large sample sizes (thousands of samples) collected from many families (normally trios but also quads) (Neale et al., 2012; De Rubeis et al., 2014; Merico et al., 2017). By comparing DNA sequences obtained from affected children to those from their parents, it is possible to identify DNMs after filtering out sequencing artifacts (Iossifov et al., 2014). This variant calling process requires a detailed bioinformatics pipeline that involves the application of different thresholds to filter for each quality parameter (Patel et al., 2014). This process could be performed following different approaches and accordingly, we can find a more or less restrictive filtering depending on the study. Nevertheless, each single DNM will finally be re-sequenced by other methods, usually Sanger sequencing, to check the accuracy of the findings. We should take into account, that the average rate of DNMs in a set of whole exome data is estimated to be in 1.2 × 10^-8^ per nucleotide per generation, and normally ASD studies have observed a similar or slightly higher rate (Conrad et al., 2011).

After this first step, all DNMs located in the coding sequence should be functionally annotated according to the impact that the predicted amino acid substitution has on protein structure and function. Thus, we can find missense DNMs and non-sense DNMs, also referred to as LoF mutations, which can in turn be classified into different subtypes: frameshift, splice site, and stop-gain. It is important to note that although LoF DNMs might be the object of greater attention, the importance of missense DNMs in ASD was recently highlighted. Therefore, such variants may produce a gain of function effect and genes carrying two or more mutations of this type were seen to be more likely to be pathogenic in ASD (Geisheker et al., 2017). Moreover, some studies have reported an overall enrichment of LoF mutations in individuals with ASD compared to their healthy relatives. In particular, heterozygous LoF mutations are present in 20% of probands but in only 10% of unaffected siblings (O’Roak et al., 2011; Neale et al., 2012; Sanders et al., 2012; Ronemus et al., 2014). Missense mutations were also more common in probands than in their siblings when larger cohorts were considered and therefore, it was calculated that missense mutations contribute to at least 10% of ASD diagnosis (Iossifov et al., 2014).

### Methods to Assess DNM Pathogenicity

Several tools can be used as functional predictors to assess DNM pathogenicity, such as Polyphen2, SIFT, CADD, and GERP (Cooper et al., 2005; Kumar et al., 2009; Adzhubei et al., 2010; Kircher, 2014). Polyphen2 is without doubt the most widely employed of these, although more recent trends prefer not to focus on just a single method but rather, to consider a combination of several *in silico* scores in order to establish criteria to classify benign and deleterious mutations (Lim et al., 2017). Indeed, an integrative approach was described not long ago that relied on a new functional genome annotation tool called Eigen. This tool provides a meta-score calculated by unifying the information obtained through several annotation methods. Therefore, Eigen provides a better discriminatory ability than other scores like CADD, SIFT, or GERP. As such, Eigen is a powerful and novel annotation tool that was successfully employed on a set of DNMs previously described in ASD and also in other psychiatric disorders like schizophrenia (Ionita-Laza et al., 2016). More recently, other measures of the deleterious nature of mutations have been developed to redefine the impact of DNMs. One of these novel scores is called, MPC (for *Missense badness, Polyphen-2* and *Constraint*), which specifically enables the deleterious effect of missense variants to be predicted. Through the use of MPC, some missense DNMs were shown to have a similar effect as LoF mutations in NDDs, information that will be extremely useful for future ASD sequencing studies (Samocha et al., 2017).

### DNMs: Relative Risk, Tolerant, and Intolerant Genes

The contribution of DNMs to the risk of ASD depends on the impact that the amino acid change in the protein coding sequence has on the protein’s behavior. Thus, the RR entailed by LoF DNMs will always be larger than that associated with missense DNMs. Moreover, both variants will provide a greater RR when they are considered jointly rather than an inherited LoF mutation alone, for example. This allows a RR to be established for each gene as a function of the class of DNM (De Rubeis et al., 2014). Moreover, some studies also consider the location of the DNM and it was shown that DNMs are more likely to occur in genome locations with a higher rate of mutation that are located close to CNVs (Merico et al., 2017). Another factor that must be taken into account when DNMs are analyzed is that there are genes that are mutation tolerant and intolerant. This means that over the entire human genome some genes are more likely to carry more functional mutations than those expected by chance (tolerant genes), while other (intolerant) genes carry fewer such mutations. Thus, DNMs found in tolerant genes are less likely to influence the development of ASD. A gene-based score RVIS has been developed that allows genes to be ranked depending on their tolerance or intolerance score (Petrovski et al., 2013; Ronemus et al., 2014). Similarly, additional information can be provided by the pLI score (*prob of being LoF intolerant*). Therefore, a gene with pLI > 0.9 is considered to be extremely LoF intolerant, and this is particularly useful when there is more than one LoF mutation in an exome and there is a need to prioritize these causal DNMs (Lek et al., 2016). The interest in this score was successfully confirmed using genetic data from NDDs, including ASD cases (Kosmicki et al., 2017).

As we can see, the discovery, identification and prioritization of DNMs and their respective ASD risk genes, requires a complex workflow. It involves several technical variables that need to be considered in order to identify the DNMs that truly influence ASD risk and to distinguish them from those that are artifacts or that are not pathogenic DNMs.

## Bioinformatics Approaches Employed in the Study of DNMs

The main aim of the bioinformatics approaches discussed in this section is to start from the genetic information obtained from the genes carrying DNMs, achieving a global vision of the related biological processes that underlie the pathogenesis of ASD (**Table 2**). As detailed below, these tools aim to integrate different sources of genetic and biological information in order to identify the biological processes underlying ASD, as well as new target genes.

**Table 2:** Bioinformatics approaches that allow WES data (genes carrying DNMs and other genetic information) to be integrated in different pathway and network analyses categorized by the input data necessary, the type of algorithm and the output results.

	Input information	Algorithm	Analysis result	Publications
TADA	DNMs (LoF > missense) + transmitted + case-control variants	Bayesian gene-based likelihood model	Prioritized list of genes depending on the impact of the mutations	De Rubeis et al., 2014 Sanders et al., 2015 Ji et al., 2016
NETBAG	Input data	Likelihood approach including a Bayesian integration of PPIs.	Identifies functional gene networks and phenotype networks	Gilman et al., 2011 Chang et al., 2014
DAWN	List of ASD genes obtained from WES studies scored by TADA	Algorithm based on the “screen and clean” principle (hidden Markov random field + FDR procedure)	Identifies gene networks that are “hot spots” within a co-expression network (RNA-seq data)	Liu et al., 2014
DAPPLE	List of ASD candidate genes	Algorithm based on permutations	Test PPIs across the genes hit by a functional DNM. Allow to redefine a huge list of putative ASD genes in a reduced but most relevant list	Neale et al., 2012 Poultney et al., 2013 Sanders et al., 2015 Parikshak et al., 2013
MAGI	List of ASD genes obtained in WES and case-control studies	Combinatorial optimization algorithm. Maximizes mutations in modules considering gene length and where DNMs are located (LoF and missense)	Creates gene clusters considering the information from PPIs and co-expression networks together	Hormozdiari et al., 2015


*Moreover, the most relevant publications employing each of them to study ASD genetics are indicated.*

### Prioritizing Novel ASD Risk Genes Carrying DNMs

The analysis of DNMs has without doubt been a step forward in the discovery of new ASD risk genes. Technically speaking, this type of analysis can only be performed on DNMs. However, it was recently shown that a more robust way to interpret WES data is to analyze DNMs together with inherited variants, given the high heritability of ASD. Therefore, other genetic variants can be added, such as SNPs from case-control studies. This approach came into use when it was seen that the proportion of ASD cases that could be explained by considering only DNMs and not other types of genetic variation was really quite small. Moreover, despite analyzing thousands of ASD cases, only tens of LoF DNMs were detected. Therefore, this combined analysis, called TADA, opened the door to expanding the list of ASD candidate genes and it made the analysis of WES data more robust (He et al., 2013; Sanders et al., 2015). This approach has been successfully employed on genetic data from the SSC and the ASC (De Rubeis et al., 2014). TADA uses a Bayesian gene-based likelihood model that weights mutations by type and mode of inheritance in this order: *de novo* LoF > *de novo* Mis3 (missense variants predicted to be damaging by Polyphen) >transmitted LoF. In this way, each DNM is given a predicted impact on the protein function. Moreover, the corresponding gene mutation rate is also considered and these categories can be extended as required for the desired analysis (He et al., 2013). Furthermore, it is possible to obtain expanded or restricted gene lists that consider the load of DNMs by gene and their predicted functional impact. This is possible because TADA generates a gene-level BF that quantifies association and its correspondence to a given FDR or *q*-value. Thus, TADA allows a prioritized list of genes to be obtained, which is perfect to use as an input for other bioinformatics tools that are optimized to create gene-networks and to unravel new related biological pathways in ASD. Recently, the TADA algorithm was modified (TADAext) allowing data from multiple populations to be employed and related NDDs to be considered together in order to discover common risk genes. As such, TADA helps define and prioritize a list of genes that can be employed as an input for additional analyses, as will be seen below (Nguyen et al., 2017).

### Gene-Network and Pathway Analysis Tools

Once gene lists are established and prioritized, several tools can be used to generate gene networks and pathways. NETBAG is one of the latest algorithms that can be successfully employed to create risk gene networks starting from information about DNMs (Gilman et al., 2011). This computational approach was also used in ASD sequencing studies to not only consider data from DNMs (SNVs and CNVs) but also, to combine this with information from other associated genomic regions identified in GWAS studies. As such, NETBAG has been successfully employed with ASD and schizophrenia data (Gilman et al., 2012). Specifically, this tool serves to establish gene clusters that identify distinct biological networks of genes, for example networks that are related to synapse development and/or neuron motility but relying on a previously described phenotype network (Gilman et al., 2011; Pinto et al., 2014). This phenotype network is based on the integration of various protein-function descriptors using Bayesian methods. The network edges will be constructed considering the likelihood that two genes participate in the same genetic phenotype (for example, ASD and/or ID). Among a list of provided genes (from each genetic study), NETBAG will create clusters of strongly connected genes by phenotype depending on the calculated likelihood (Chang et al., 2014). Therefore, the most important characteristic of NETBAG is that the underlying network is created by sets of genes previously associated with ASD and/or ID phenotypes. Once these clusters are formed, specific biological processes related to each one can be added integrating GO, KEGG, and PPI descriptors. Another algorithm that could be very helpful in the search for ASD risk genes and that helps to integrate DNM information, is DAWN. DAWN works in conjunction with a network analysis tool like TADA that sets a score for each gene, and it can identify hotspots (clusters of strong scores) among the complex gene networks that can be established when the whole set of TADA genes is considered. This algorithm works through a hidden Markov random field, a generalization of a hidden Markov model that is widely employed when modeling biological processes. The particular strength of DAWN is that it relies on another type of information to build these new clusters, integrating transcriptomic data (RNA-seq) analyzed using a WGCNA approach (a method that will be discussed later in more detail). Once the large co-expression network is created, DAWN will help to identify clusters of strongly correlated genes. Therefore, using the TADA scores obtained previously, DAWN will identify ASD risk genes, always performing a multiple testing correction (FDR). DAWN can also incorporate any additional variables as transcription targets if one or more key transcription factor were meaningful to the analysis (Liu et al., 2014, 2015). Therefore, DAWN works in conjunction with TADA but while it is TADA that prioritizes genes carrying DNMs, DAWN moves a step forward by creating gene networks and subnetworks that help to detect novel genes that would not be revealed by using TADA alone. Indeed, DAWN uses TADA scores for different sets of previously published genes. For example, *GRIN2B* is an ASD risk gene reported to be a carrier of multiple LoF mutations (TADA *q*-value 0–0.0025). Consequently, DAWN can establish *ACTN2*, *DLG1*, *CBL*, *AP2A1*, and *DLG4* among others as novel *GRIN2B* connectors, assigning them to a cluster of receptor signaling and protein scaffolding genes (O’Roak et al., 2011; Liu et al., 2014).

Another two complementary strategies that are commonly used in these types of studies are enrichment analysis and PPI networks. GSEA serves to classify genes that are over-represented in a large dataset, identifying those groups significantly enriched or depleted according to another source of external information (e.g., GO terms, KEGG terms, expression data…) and thereby helping to identify a variety of biological signatures among them (Wen et al., 2016). There are several tools and databases that allow GSEA analysis to be run, and one of the most commonly employed is that provided by the Broad Institute website in cooperation with MSigDB. This specific GSEA tool was successfully run in large gene sets like those reported by SFARI, an evolving online database which contains up-to-date information of genes associated to ASD^1^. In addition, hypergeometric distribution can be employed to examine how SFARI genes and other gene sets (GO terms, KEGG) overlap. This tool has led to the characterization of several pathways functionally associated in ASD, such as calcium and MAPK signaling pathways (Wen et al., 2016).

Another GSEA tool is DAVID, an enrichment analysis tool that was employed in ASD genetic studies (Dennis et al., 2003). DAVID is commonly used to consider how informative a gene list obtained from genetic studies is about ASD etiology (Pinto et al., 2014). Thus, DAVID can discover groups of functional-related genes by using different libraries (GO terms for example) to help identify the enrichment of different biological processes from an extended gene list (Huang et al., 2008, 2009; Sanders et al., 2015). Therefore, DAVID and GSEA both allow enriched functionally related gene groups to be discovered and thus, both tools are applied indistinctly for the purpose of ascribing general biological functions to genes. However, DAVID also features some additional options, and it is able to highlight functional protein domains and motifs in those relevant genes.

Another GSEA tool is Enrichr, currently one of the most comprehensive tools that not only includes GO ontologies but also, new gene libraries like target microRNAs, LINCS libraries and even epigenetic data from the RoadMap Epigenomics Project. Moreover, Enrichr also allows the GSEA results to be exported, whether networks, tables or bar graphs, which can be sorted by *p*-values, *q*-values or z-scores for the different terms analyzed (Wen et al., 2016).

The use of PPIs is another strategy that helps to integrate additional information from a different biological hierarchy. PPI data are crucial to define how proteins interact in cellular processes and also, to identify others that could be connected in order to construct an interaction map (McDowall et al., 2009). There are several PPI databases available like BioGRID, STRING, MINT, KEGG, DIP, HPRD, or IntACt (Lehne and Schlitt, 2009). Therefore, ASD genes of interest can be mapped against these PPI networks, identifying connected genes that have not been found previously, or highlighting previously weakly associated ASD genes. Moreover, this approach allows gene sub-networks to be redefined whose involvement in ASD has previously been reported (Corominas et al., 2014). The ultimate aim would be to organize this information to create gene clusters, each of them characterized by cellular processes (Liu et al., 2014). DAPPLE is an algorithm frequently employed in genetic studies of ASD that works using PPI networks. Specifically, DAPPLE searches significant physical interactions between proteins encoded by genes associated with ASD. Moreover, it allows additional genes that have been reported in other independent studies to be introduced in order to expand the interaction network. The perfect strategy is to seed together the interaction network built by DAPPLE with data obtained from several available PPI databases, expanding the known information with new nodes and connectors (Rossin et al., 2011; Neale et al., 2012; Poultney et al., 2013).

Therefore, GSEA allows gene sets to be functionally annotated with their corresponding biological terms and significantly enriched or depleted groups of genes to be identified. However, PPIs represent another source of biological information that can be integrated into bioinformatics tools like DAPPLE, expanding the interaction network to include novel genes.

### Characterization of the Biological Processes Underlying ASD Pathogenesis

As explained before, ASD is an extremely heterogeneous disorder, characterized by its genetic variability. It is expected that around 1,000 genes are involved in ASD, meaning that no one gene is likely to explain more than 1% of cases (De Rubeis et al., 2014), which makes functional studies difficult and complicates the identification of high value targets for treatments. One possible solution to help resolve this problem is to look for the common biological mechanisms that could be disrupted in a recurrent manner through the use of integrative systems biology approaches, such as those described in the previous section (Parikshak et al., 2015).

Initial studies focused on testing if the genes disrupted by truncating mutations converge and are related to previously reported ASD genes. Therefore, it is expected that those genes that interact significantly also share common functions and are probably involved in the same biological pathways (Uetz et al., 2000). A PPI network was constructed based on the data collected by GeneMANIA, considering a list of genes carrying severe mutations (Mostafavi et al., 2008; O’Roak et al., 2012). As such, it was demonstrated that 39% of genes carrying truncating mutations directly interact in this network. This physical interaction between genes is an indicator of their implication in some common biological mechanisms that could underlie ASD pathogenesis. Therefore, those genes carrying truncating mutations are ranked higher. This study is a perfect example of how information about DNMs can be used to identify other potential ASD risk genes using the correct tools and methods, helping to map those interconnected genes in the corresponding biological processes. In this case, the main biological network revealed was a β-catenin/chromatin remodeling protein network (O’Roak et al., 2012).

**FIGURE 1:**
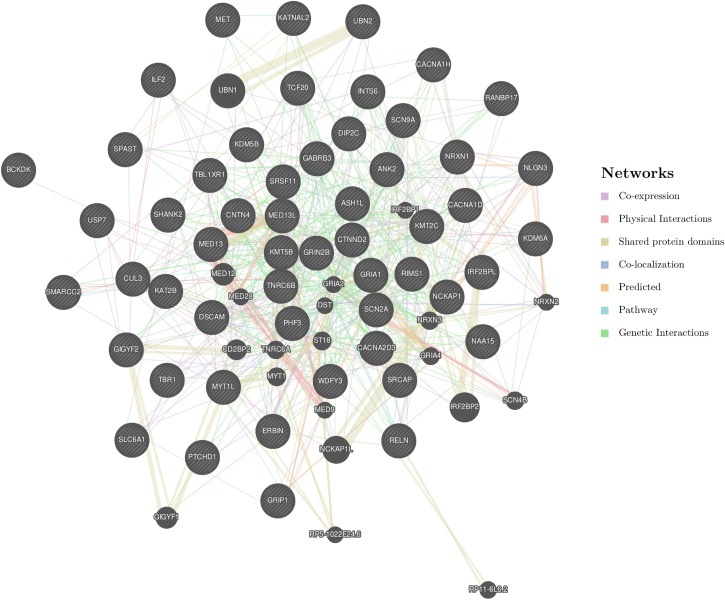
GeneMANIA network created from 54 SFARI genes with scores 1 or 2. The following genes were used as the input: *SPAST*, *CUL3*, *KMT2C*, *NCKAP1*, *RIMS1*, *SRCAP*, *TCF20*, *TNRC6B*, *INTS6*, *BCKDK*, *MET*, *MED13*, *KMT5B*, *ERBIN*, *KAT2B*, *ASH1L*, *SRSF11*, *KDM5B*, *PHF3*, *IRF2BPL*, *MED13L*, *SCN2A*, *TBR1*, *SMARCC2*, *ILF2*, *CNTN4*, *ANK2*, *KDM6A*, *DIP2C*, *GRIA1*, *GRIP1*, *SLC6A1*, *CACNA1D*, *CACNA2D3*, *UBN2*, *SHANK2*, *WDFY3*, *NAA15*, *PTCHD1*, *GABRB3*, *KATNAL2*, *SCN9A*, *CTNND2*, *DSCAM*, *TBL1XR1*, *NRXN1*, *MYT1L*, *USP7*, *RELN*, *NLGN3*, *CACNA1H*, *GIGYF2*, *RANBP17*, and *GRIN2B*. These genes are indicated with stripes. Moreover, another 20 strongly connected genes that were detected by GeneMANIA are also represented.

We performed a similar analysis but choosing only those ASD risk genes carrying DNMs from previous studies and collected in the SFARI database with scores of 1 and 2 (high-confidence and strong candidate genes) (**Supplementary Table 1**). Therefore, 54 genes were used as input in GeneMANIA, revealing 20 related genes and 681 links between them (**Figure 1**). In order to create this network, GeneMANIA employs data from co-expression experiments but also physical interactions, shared protein domains, co-localization and previously reported genetic interactions. Each gene–gene interaction is given a weight and assigned to a corresponding network group (**Supplementary Table 2**). The biological functions of these genes and their corresponding FDRs are also obtained (**Supplementary Table 3**), revealing them to be: neuron cell–cell adhesion, vocalization behavior, glutamate receptor signaling pathway, cognition, and neuron projection.

It should be noted that methodological improvements have allowed genes affected by DNMs and *de novo* CNVs to be included in the same study, leading to the consideration of a higher percentage of ASD heritability. Therefore, these genes cluster together in networks enriched in different biological functions, such as synaptic function, neuronal signaling, channel activity, and chromatin modification (Gilman et al., 2012; Pinto et al., 2014). The same pathways were also identified in subsequent studies, confirming the important role of these processes in ASD neurobiology (De Rubeis et al., 2014; Krishnan et al., 2016).

Accordingly, many of the ASD genes characterized are synaptic genes, including *NLGN3* and *NLGN4X* (Jamain et al., 2003), *SHANK3* (Durand et al., 2006), *NRXN1* (Autism Genome Project Consortium et al., 2007) and *CNTNAP2* (Arking et al., 2008). Therefore, both the development and maintenance of synaptic contacts appear to be a key factor in ASD pathogenesis. Conversely, chromatin regulation also influences neural development and during this process, many events must be precisely orchestrated and mis-regulation can result in cognitive deficits. The modification of chromatin structure controls cell fate and function (van Bokhoven, 2011; Jakovcevski and Akbarian, 2013; Ronan et al., 2013) and dozens of chromatin remodelers have been implicated in ASD and other neurological diseases, including Coffin-Siris syndrome (Tsurusaki et al., 2012), Nicolaides-Baraitser syndrome (Van Houdt et al., 2012), CHARGE syndrome (Vissers et al., 2004), or Rubinstein-Taybi syndrome (Roelfsema et al., 2005). Some of the best studied genes belongs to the CHD. Indeed, functional studies in mice have shown that CHD5 and CHD8 haploinsufficiency causes morphological changes in the brain and behavioral symptoms consistent with ASD (Pisansky et al., 2017; Platt et al., 2017).

A representation of this vast list of ASD genes discovered through the identification of DNMs and those biological processes in which they are involved (see **Supplementary Table 1**) provides a representative gene-list taken from the SFARI database as well as useful additional information.

Another important group of genes overrepresented in ASD networks are FMRP targets, which are defined as gene encoding transcripts that bind to FMRP (Iossifov et al., 2012). This set of genes includes *NLGN1*, *NRNK1*, *SHANK 3*, *PTEN*, *TSC2*, and *NF1*, and it overlaps with the list of candidate ASD genes from the SFARI database (Darnell et al., 2011) that mainly encode synaptic proteins, transcription factors and chromatin modifiers (Korb et al., 2017).

## Correlation of DNMs With Gene Expression in Co-Expression Networks

Gene co-expression networks (GCNs) represent another tool commonly used in ASD studies. The key point of this approach is to construct gene networks considering not only the genetic data obtained in WES studies but also, to correlate this information with expression data from RNA-seq experiments. Thus, these gene networks allow different temporal-spatial modules to be identified based on expression at different developmental stages and in different brain areas (van Dam et al., 2017). As such, it is possible to achieve the ultimate goal of understanding the genetic causes of ASD and to relate this to gene regulation at different levels. Such information permits the role of DNMs in the pathogenesis of ASD to be better understood, helping to define the molecular pathways and the neural circuits that affect cognition and behavior. Therefore, this complex analytical approach will ultimately construct a spatiotemporal co-expression network of ASD genes.

The generation of co-expression networks involves the application of different statistical approaches, although two main steps are critical and always considered by the corresponding algorithms: calculation of a measure of co-expression (for which different mathematical methods could be used); and the establishment of a significance threshold (Song et al., 2012).

WGCNA constructs networks by using the default Pearson correlation. WGCNA find modules of expression of highly correlated genes and it identifies eigengenes for each module. For this, WGCNA employs a PCA to extract the most representative part of the expression data. Therefore, each module (given by an expression value) corresponds to an eigengene and these eigengenes can be employed to construct the related biological networks.

In addition to WGCNA, other methods were recently employed to analyze ASD genomic data, such as MAGI, which represents a further step-forward in the use of this type of tool (**Table 2**). MAGI not only allows expression data (RNA-seq) to be integrated with genetic information (from *missense* or *LoF* mutations to case-control studies) but also, representative biological information from PPIs can also be added (Leiserson et al., 2015). This data integration was successfully employed with WES data from ASD and ID, facilitating the identification of two differentiated modules of genes during brain development, one expressed from 8–14 weeks post-conception, which includes genes related to the Wnt pathway, and another that contains genes related to synaptic function and that is more strongly expressed in postnatal stages (Hormozdiari et al., 2015). The vast majority of ASD co-expression networks have employed the data available at BrainSpan^2^, which includes RNA-seq data from sixteen targeted cortical and subcortical structures at different stages of human brain development (prenatal and postnatal development) (Kang et al., 2011).

Expression in brain tissues has been analyzed in different studies, integrating this data with that obtained in genetic studies to identify at which developmental stages and in which brain areas both sources of information overlap. *Post-mortem* brain tissue samples (cases and controls) were analyzed to identify which ASD genes are altered in specific regions. WGCNA was applied to these data to integrate the differences in expression between cases and controls in a systems biology context. Two network modules were enriched in genes highly correlated with ASD: one for genes down-regulated in ASD patients, showing functional enrichment for some GO terms like synaptic function, vesicular transport and neuronal projection; the other containing up-regulated genes with an enrichment of the immune and inflammatory GO categories. The integration of genetics data with co-expression modules has shown that the former may identify potential causes of ASD, while the latter suggests the biological response (Voineagu et al., 2011). Subsequently, a RNA-seq study was performed on a larger ASD cohort, demonstrating similar results. Therefore, altered neural activity and an enhanced microglial response was proposed in ASD brains, highlighting the role of the immune system and synapses in ASD (Gupta et al., 2014). However, the largest cohort of brain samples analyzed to date identified 24 co-expression modules after WGCNA analysis with RNA-seq data. Six modules were associated with ASD, three down-regulated and three up-regulated. Synaptic and neuronal genes were found among the down-regulated modules, while glial function and biological pathways related to inflammatory processes were enriched in the up-regulated modules. Moreover, one of the 24 modules was enriched in DNMs previously associated with ID, while another module was enriched for lncRNAs (Parikshak et al., 2016).

Co-expression networks constructed from publicly available datasets have revealed how ASD genes are differentially expressed during early, mid and late fetal development, indicating that they are directly involved in the development of the prefrontal, temporal, and cerebellar cortex (Willsey et al., 2013; Chang et al., 2014; Krishnan et al., 2016). In particular, strongly associated ASD genes converge in glutamatergic projection neurons located in layers 5 and 6 of human mid-fetal prefrontal and primary motor somatosensory cortex (Willsey et al., 2013). A WGCNA analysis employing an enrichment strategy produced a list of genes from SFARI that mapped into different expression modules (Parikshak et al., 2013). This allowed these genes to be traced to specific neurodevelopmental stages and neuronal cell types. Therefore, the integration of expression data allows ASD risk genes carrying DNMs (and/or other genetic variants) to be correlated with a superior hierarchical level of biological information, expanding our understanding of ASD pathogenesis. Through such studies at the circuit level, ASD genes have been seen to be enriched in glutamatergic neurons in upper cortical layers. It is worth noting that this result is different from the findings obtained in the previous study in which ASD genes converged in layer 5/6 cortical projection neurons. Therefore, these genes converged in modules associated with biological functions like early synaptic development and transcriptional regulation. Interestingly, both modules were enriched in targets of the FMRP gene, indicating that translational regulation could be a link between molecular pathways that are co-expressed during fetal cortical development (Parikshak et al., 2013). Alternatively, a spatial analysis revealed that the activity of ASD genes is widely distributed throughout the brain, which is consistent with the broad spectrum of symptoms associated with ASD. However, some specific areas were apparently more strongly linked to ASD, such as the cerebellum, striatum, amygdala, and thalamus (Chang et al., 2014; Krishnan et al., 2016).

A recent study using co-expression networks and enrichment approaches allowed different types of DNMs to be studied (Shohat et al., 2017). Moreover, different patterns of expression were described in the brain for genes associated with different neuropsychiatric disorders. Enrichment analysis of protein coding genes mapped to those previously described WGCNA modules (Parikshak et al., 2013) in different brain areas and at distinct neurodevelopmental stages. In addition to ASD genes, genes carrying mutations associated with schizophrenia and ID were also tested. Accordingly, genes carrying LoF DNMs in ASD and ID were found to be preferentially expressed in the fetal brain (cortex) and they were related to chromatin organization. However, genes carrying missense DNMs were associated with schizophrenia and they were active in the young adult cortex during adolescence (Parikshak et al., 2013). Therefore, these approaches appear to be able differentiate distinct biological pathways that are associated with ASD, schizophrenia and ID (Shohat et al., 2017).

## Paternal Age and DNMs

A relationship between advanced paternal age and increased ASD risk has been established in different studies (de Kluiver et al., 2016; Janecka et al., 2017). Multiple biological mechanisms can explain this relationship, not only DNMs but also epigenetic changes associated with aging (Atsem et al., 2016). DNMs are typically present in the sperm or egg of one parent and they are then transmitted to the embryo. Thus, these mutations are present in all cells within the offspring. Interestingly, WES data enables the paternal or maternal origin of DNMs to be determined, identifying which parental haplotype carries the same mutation as that found in the proband. Interestingly, it was noted that most of DNMs originate in the father (Iossifov et al., 2012; O’Roak et al., 2012), which may perhaps not be surprising given the ratio in the number of spermatozoa to eggs produced. In addition, the number of DNMs is positively correlated with paternal age and it has been calculated that each additional year of paternal age at the moment of conception results in two extra DNMs in the proband. Conversely, the number of mutations transmitted maternally remains relatively constant over the years (Kong et al., 2012). The number of cell divisions that male germ cells continuously suffer could possibly explain these findings, while female eggs do not actively divide during the female’s reproductive years (Crow, 2000). Together, these results are consistent with a hypothesis in which a higher paternal age entails an increased ASD risk in probands due to the higher rate of mutations.

Nevertheless, although the biological hypothesis plausibly explains the relationship between paternal age and ASD risk, it is unlikely to reveal more than a modest genetic risk fraction (10–20%; Gratten et al., 2016). Therefore, there are additional mechanisms to be considered, especially taking into account that offspring of younger parents are also at risk of some mental disorders (McGrath et al., 2014). One alternative hypothesis suggests that delayed fatherhood is correlated with a tendency toward neuropsychiatric illnesses. Therefore, genetic risk factors for psychiatric disorders that are highly heritable may be shared by older fathers and their offspring (Gratten et al., 2016). Both hypotheses are not mutually exclusive and they reflect how the relationship between risk and paternal age is probably due to a complex interrelated matrix of epidemiological and genetic factors.

## Post-Zygotic Mutations (PZMs) and Mosaicism in ASD

**FIGURE 2:**
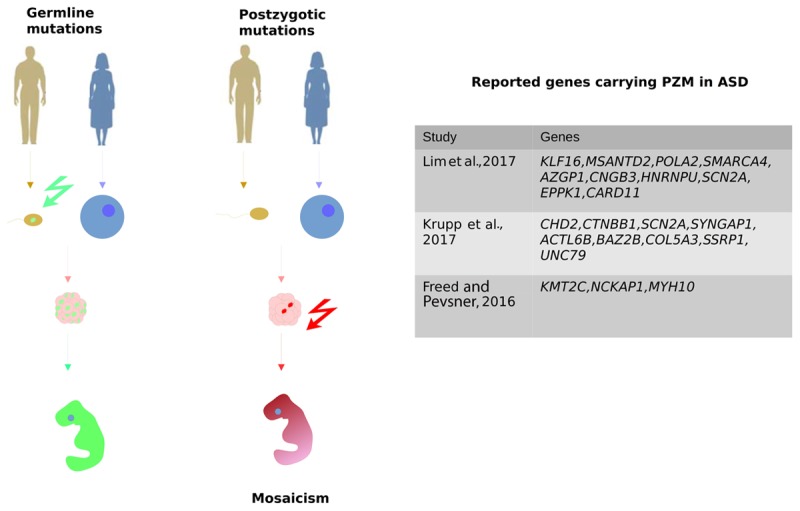
Post-zygotic mutation (PZMs) are acquired after the zygote forms, as opposed to germline mutations that are inherited from the parents. Therefore, PZMs are not present in every cell of the organism, which is therefore a mosaic individual. It was recently demonstrated that PZMs contribute significantly to ASD risk. The most relevant studies focusing on the detection of PZMs are represented along with the genes seen to carry different PZMs.

PZMs are another type of DNMs that are beginning to generate much interest in ASD genetic studies. PZMs occur during the mitotic cell divisions that generate the embryo after fertilization and as a result, a mosaic individual is created in which a variable number of cells carry the mutation (**Figure 2**; Biesecker and Spinner, 2013). As such, the developmental timing and cell lineages affected will probably determine the severity of the symptoms in these disorders. PZMs are implicated in several brain disorders, including epilepsy, cortical malformations, or RASopathies (Kurek et al., 2012; Lee et al., 2012; Poduri et al., 2013; Jamuar et al., 2014). Indeed, it was shown that some PZMs carried by the X-Linked methyl CpG binding protein 2 (*MECP2*) gene cause Rett’s Syndrome. Rett’s syndrome is usually lethal in males and dominant in females but in some cases, mosaic mutations have been reported that are compatible with male viability (Pieras et al., 2012).

The detection of PZMs has been a challenge because they are tissue-specific and ASD brain tissue is almost never available. In order to solve this problem, sensitive genotyping techniques are necessary, such as SNP microarrays, NGS and WES studies. The success of these technologies relies on the ability to analyze a large number of cells at once, which helps to increase the probability of detecting mutations in a mosaic state. SNP arrays can detect mosaics when at least 5% of the cells of an individual are carrying the mutation (Conlin et al., 2010), while NGS can also detect mosaic mutations based on the fraction of unusual alleles calculated through the AAF. NGS provides deep sequencing coverage that allows for the observation of a sufficient number of reads with reference and alternate alleles to accurately calculate AAF. In this context, PZMs have been reported when the AAF ≤ 40%, shifting from the 50:50 ratio expected for heterozygous germline mutations. Therefore, the deep sequencing coverage of panels of candidate genes allow mutations to be detected that are present in at least 5% of the reads, meaning that 10% of the cells in the individual carry the variant (Jamuar et al., 2014). WES is also sensitive enough to detect PZMs when the AAF is at least 15%, which means that mutations are present in about 25–30% of the cells (Pagnamenta et al., 2012; Genovese et al., 2014).

Despite the potential role of PZMs in the etiology of ASD, the common variant calling pipelines employed in WES lose this valuable source of information due to the application of strict filters to avoid artifacts. Reanalysis of the SSC using novel calling approaches to specifically characterize SNVs that are likely to be PZMs led to a higher proportion of mosaic SNVs (22%) than those reported previously (Krupp et al., 2017). Elsewhere, when WES data was recalled from the same cohort, about 80% of the PZMs detected had not been published before (Lim et al., 2017). Indeed, those variants were validated using three different techniques, proving that PZMs can be better detected by modifying the current pipelines (**Table 3**). In addition, these studies identified PZMs in high-confidence NDD risk genes, such as *SCN2A*, *CTNNB1*, *SYNGAP1*, and *HNRNPU*, evidence that at least a proportion of PZMs predispose to ASD. Moreover, new candidate genes were significantly enriched in PZMs, such as *KLF16* and *MSANTD2* (**Figure 2**).

**Table 3:** Results of the two main studies analyzing PZMs in ASD cohorts.

Study	Krupp et al., 2017	Lim et al., 2017
Number of families analyzed	2264	5947
% Of PZMs detected applying new bioinformatics pipelines	22%	9.7%
% Of mutations not previously published	70.64%	83.3%


*Both of them reanalyzed previously published data but applying different bioinformatics pipelines in order to detect PZMs involved in ASD.*

Detailed analysis of these variants, especially the truncating mutations, revealed novel and uncharacterized pathways and cellular processes that may possibly be involved in ASD pathogenesis (Lim et al., 2017). Surprisingly, an increased burden of synonymous PZMs in probands has been reported, with synonymous mutations enriched in splice sites, indicating that splicing regulation could contribute to ASD pathogenesis. Moreover, around 2.3% of ASD simplex cases harbor a synonymous PZM related to ASD risk. However, missense and LoF PZMs were also associated with ASD, most of them affecting genes expressed in the brain and other high confidence ASD risk genes. Thus, it was estimated that PZMs contribute about 4% to the overall architecture of ASD (Krupp et al., 2017; Lim et al., 2017). The spatiotemporal distribution of these mutations has also been reported, pointing to the amygdala as a brain area of interest that merits further attention in terms of ASD pathogenesis.

In conclusion, preliminary studies have produced strong evidence of the importance of considering PZMs in ASD genetic studies. Therefore, it is necessary to elucidate how PZMs contribute to ASD (and other NDDs), determining the genetic risk that could be explained by them. Thus, different analytical approaches and study designs need to be developed, involving larger cohorts than those analyzed previously and developing improved variant detection pipelines for PZMs.

## Caveats and Future Perspectives in the Study of DNMs and ASD Genetics

Despite the important advances made in the study of ASD genetics over recent years, some caveats still exist regarding the detection of DNMs, which will hopefully be resolved by future studies. The study of PZMs carried out by the ASC has helped establish an emergent type of genetic variation that had been dismissed until now (Lim et al., 2017). Subsequently, other studies have focused on this interesting and informative type of DNM (Krupp et al., 2017), although the filtering and variant calling processes used in these studies are quite different, highlighting the need for a single, optimized and unified pipeline. This is without doubt one of the future areas that will benefit from further research. In relation to this, a proportion of *de novo* CNVs are also expected to be postzygotic, yet the repercussion of this type of post-zygotic structural variation in ASD genetic architecture has still to be studied in detail. This will require the implementation of suitable and valid bioinformatics pipelines. Likewise, huge public repositories should be reanalyzed following different pipelines in order to detect PZMs that may have been missed until now, for example the SSC that currently contains 8975 whole genomes. Such efforts will help to highlight new genetic factors involved in ASD pathogenesis.

Another relevant area of study involves the proportion of DNMs in children that are parental mosaic mutations, asymptomatic in the parents yet transmitted to the offspring. The existence of this biological phenomenon was well documented in other genetic diseases and in fact, a genetic test to detect parental mosaicism is included in some routine diagnostic tests (Campbell et al., 2014; Frederiksen et al., 2015). In terms of ASD genetics, the overall incidence of parental somatic mosaicism reported to date is extremely low (6.8% of all DNMs), yet not inexistent (Dou et al., 2017; Krupp et al., 2017). Therefore, future studies on the largest possible number of families, employing different variant detection methods, will be decisive to elucidate the exact role of parental mosaic DNMs in ASD. The identification of genes carrying PZMs and the development of a genetic diagnosis through a simple blood test in parents will also require further research.

There is another type of genetic variation that will require the development of new detection methods for indels (De Rubeis et al., 2014; Brandler et al., 2016). *De novo* indels were previously associated with ASD (*KMT2E* and *RIMS1*) but the systematic analysis of disrupting indels will require the development of robust and more accurate methods (Dong et al., 2014). Therefore, it was demonstrated that the detection of indels could be enhanced by using new algorithms that allow the assembly of DNA sequences to be redefined in order to detect them more accurately. Indeed, through the analysis of samples from the SSC it was demonstrated that disrupting *de novo* indels plays a major role in ASD genetics (Narzisi et al., 2014).

*De novo* mutations in non-coding regions have become of interest in recent years. Previous WES studies were unable to detect these variants due to the lack of coverage and sequencing depth across non-coding regions (promoter and regulatory regions). However, there is evidence that ASD genes harbor hotspots of hypermutability in non-coding regions and besides, deleterious mutations across them are subjected to strong negative selection just like the LoF mutations located in the coding region (Michaelson et al., 2012; Warr et al., 2015). Studying non-coding regions demonstrated that promoter regions with *in vivo* enhancer activity in the central nervous system are enriched in DNMs (Turner et al., 2017). The important role of DNMs in NDDs was also demonstrated by targeted sequencing of some selected types of promoter regions, showing that around 1–3% of patients with no genetic diagnosis carry pathogenic DNMs in some of these regions (Short et al., 2018). Another recent study reported rare SVs located in *cis*-regulatory elements of intolerant genes and their inheritance from parents may contribute to ASD in about 0.77% of cases (Brandler et al., 2018). Moreover, when the role of *de novo* SVs (∼5.1%) was assessed, the importance of these variants for future studies was evident. Recently, novel analytic pipelines were developed to integrate DNM information from non-coding and coding regions to characterize the broad spectrum of ASD genetic variability, with non-coding *de novo* indels giving more significant results than those expected by chance (Werling et al., 2018).

These data highlight the current need to perform ASD genetic studies using WGS instead of traditional exome studies. As such, the effort of the SSC in bringing together almost 8975 whole genomes for genetic analysis, including fathers, mothers, affected and unaffected siblings, is noteworthy (Ku et al., 2012; Lelieveld et al., 2015).

Regarding the integration of DNM information into higher biological hierarchies using gene and protein networks, it is also expected that new bioinformatics approaches will shortly allow the implementation of integrative analysis frameworks adapted to ASD biology. These integrative analyses will not only take into account high-throughput data from gene expression and PPI networks but also epigenetic data, information on microRNA regulation, splicing events and even quantitative trait loci when gene information from SNPs is considered together with DNM data. This huge amount of biological information will help define a more detailed and valid map of the neurobiological pathways involved in ASD.

## Conclusion

Studies into ASD genetics and specifically, DNMs have come a long way in the last few years. However, there are still some gaps to be filled that will require further analysis and the development of novel bioinformatics approaches to tackle them in sufficient detail. The ultimate goal will be to obtain the most complete and detailed biological map of ASD described to date, a map integrating genetic information with other complementary omics data, in order to unravel the complex gene networks and cellular pathways involved in ASD.

## Author Contributions

AA-G and CR-F wrote the paper. AC critically revised the work and approved the final content. AA-G, CR-F, and AC participated in the design and coordination of the review.

## Conflict of Interest Statement

The authors declare that the research was conducted in the absence of any commercial or financial relationships that could be construed as a potential conflict of interest.

## Footnotes

1 https://gene.sfari.org/

2 http://www.brainspan.org

## Abbreviations

AAF: alternate allele frequency
ASC: Autism Sequencing Consortium
ASD: Autism Spectrum Disorder
BF: Bayes factor
CADD: combined annotation dependent depletion
CHD: chromodomain helicase DNA-binding family
CNV: copy number variation
DAPPLE: Disease Association Protein–Protein Link Evaluator
DAVID: Database for Annotation, Visualization and Integrated Discovery
DAWN: Detecting Association with Networks
DNM: *de novo* mutation
DZ: dizygotic
FDR: false discovery rate
GCNs: gene co-expression networks
GERP: genomic evolutionary rate profiling
GO: gene ontology
GSEA: gene set enrichment analysis
GWAS: genome-wide association study
ID: intellectual disability
LoF: loss of function
MAF: minor allele frequency
MAGI: merging affected genes into integrated networks
MPC: Missense badness, Polyphen-2 and constraint
MsigDB: molecular signatures database
MZ: monozygotic
NDD: neurodevelopmental disorder
NETBAG: NETwork-based analysis of genomic variation
NGS: next generation sequencing
PCA: principal component analysis
PGC: Psychiatric Genomic Consortium
pLI: prob of being LoF intolerant
PPI: protein–protein interaction
PZM: post-zygotic mutation
RR: relative risk
RVIS: Residual Variation Intolerance Score
SFARI: Simons Foundation Autism Research Initiative
SNP: single nucleotide polymorphism
SNV: single nucleotide variations
SSC: Simons Simplex Collection
SV: structural variant
TADA: transmission and *de novo* association test
WES: whole exome sequencing
WGCNA: weighted correlation network analysis
WGS: whole genome sequencing

## References

[B1] Adzhubei I. A. Schmidt S. Peshkin L. Ramensky V. E. Gerasimova A. Bork P. (2010). A method and server for predicting damaging missense mutations. *Nat. Methods* 7 248–249. 10.1038/nmeth0410-248 20354512 PMC2855889

[B2] American Psychiatric Association (2013). *Diagnostic and Statistical Manual of Mental Disorders.* Arlington, VA: American Psychiatric Publishing 10.1176/appi.books.9780890425596

[B3] Anney R. Klei L. Pinto D. Regan R. Conroy J. Magalhaes T. R. (2010). A genome-wide scan for common alleles affecting risk for autism. *Hum. Mol. Genet.* 19 4072–4082. 10.1093/hmg/ddq307 20663923 PMC2947401

[B4] Arking D. E. Cutler D. J. Brune C. W. Teslovich T. M. West K. Ikeda M. (2008). A common genetic variant in the neurexin superfamily member CNTNAP2 increases familial risk of autism. *Am. J. Hum. Genet.* 82 160–164. 10.1016/j.ajhg.2007.09.015 18179894 PMC2253968

[B5] Atsem S. Reichenbach J. Potabattula R. Dittrich M. Nava C. Depienne C. (2016). Paternal age effects on sperm *FOXK1* and *KCNA7* methylation and transmission into the next generation. *Hum. Mol. Genet.* 25 4996–5005. 10.1093/hmg/ddw328 28171595 PMC5418740

[B6] Autism Genome Project Consortium Szatmari P. Paterson A. D. Zwaigenbaum L. Roberts W. Brian J. (2007). Mapping autism risk loci using genetic linkage and chromosomal rearrangements. *Nat. Genet.* 39 319–328. 10.1038/ng1985 17322880 PMC4867008

[B7] Autism Spectrum Disorders Working Group of The Psychiatric Genomics Consortium (2017). Meta-analysis of GWAS of over 16,000 individuals with autism spectrum disorder highlights a novel locus at 10q24.32 and a significant overlap with schizophrenia. *Mol. Autism* 8:21. 10.1186/s13229-017-0137-9 28540026 PMC5441062

[B8] Bailey A. Le Couteur A. Gottesman I. Bolton P. Simonoff E. Yuzda E. (1995). Autism as a strongly genetic disorder: evidence from a British twin study. *Psychol. Med.* 25 63–77. 10.1017/S0033291700028099 7792363

[B9] Betancur C. (2011). Etiological heterogeneity in autism spectrum disorders: more than 100 genetic and genomic disorders and still counting. *Brain Res.* 1380 42–77. 10.1016/j.brainres.2010.11.078 21129364

[B10] Biesecker L. G. Spinner N. B. (2013). A genomic view of mosaicism and human disease. *Nat. Rev. Genet.* 14 307–320. 10.1038/nrg3424 23594909

[B11] Brandler W. M. Antaki D. Gujral M. Kleiber M. L. Whitney J. Maile M. S. (2018). Paternally inherited cis-regulatory structural variants are associated with autism. *Science* 360 327–331. 10.1126/SCIENCE.AAN2261 29674594 PMC6449150

[B12] Brandler W. M. Antaki D. Gujral M. Noor A. Rosanio G. Chapman T. R. (2016). Frequency and complexity of de novo structural mutation in autism. *Am. J. Hum. Genet.* 98 667–679. 10.1016/j.ajhg.2016.02.018 27018473 PMC4833290

[B13] Buxbaum J. D. Daly M. Devlin B. Lehner T. Roeder K. State M. (2013). The autism sequencing consortium: large scale, high throughput sequencing in autism spectrum disorders. *Neuron* 76 1052–1056. 10.1038/nmeth.2250.Digestion PMC3863639 23259942

[B14] Campbell I. M. Yuan B. Robberecht C. Pfundt R. Szafranski P. McEntagart M. E. (2014). Parental somatic mosaicism is underrecognized and influences recurrence risk of genomic disorders. *Am. J. Hum. Genet.* 95 173–182. 10.1016/j.ajhg.2014.07.003 25087610 PMC4129404

[B15] Chang J. Gilman S. R. Chiang A. H. Sanders S. J. Vitkup D. (2014). Genotype to phenotype relationships in autism spectrum disorders. *Nat. Neurosci.* 18 191–198. 10.1038/nn.3907 25531569 PMC4397214

[B16] Conlin L. K. Thiel B. D. Bonnemann C. G. Medne L. Ernst L. M. Zackai E. H. (2010). Mechanisms of mosaicism, chimerism and uniparental disomy identified by single nucleotide polymorphism array analysis. *Hum. Mol. Genet.* 19 1263–1275. 10.1093/hmg/ddq003 20053666 PMC3146011

[B17] Conrad D. F. Keebler J. E. M. Depristo M. A. Lindsay S. J. Zhang Y. Casals F. (2011). Variation in genome-wide mutation rates within and between human families. *Nat. Genet.* 43 712–714. 10.1038/ng.862 21666693 PMC3322360

[B18] Cooper G. M. Stone E. A. Asimenos G. Green E. D. Batzoglou S. Sidow A. (2005). Distribution and intensity of constraint in mammalian genomic sequence. *Genome Res.* 15 901–913. 10.1101/gr.3577405 15965027 PMC1172034

[B19] Corominas R. Yang X. Lin G. N. Kang S. Shen Y. Ghamsari L. (2014). Protein interaction network of alternatively spliced isoforms from brain links genetic risk factors for autism. *Nat. Commun.* 5:3650. 10.1038/ncomms4650 24722188 PMC3996537

[B20] Crow J. F. (2000). The origins, patterns and implications of human spontaneous mutation. *Nat. Rev. Genet.* 1 40–47. 10.1038/35049558 11262873

[B21] Darnell J. C. Van Driesche S. J. Zhang C. Hung K. Y. S. Mele A. Fraser C. E. (2011). FMRP stalls ribosomal translocation on mRNAs linked to synaptic function and autism. *Cell* 146 247–261. 10.1016/j.cell.2011.06.013 21784246 PMC3232425

[B22] de Kluiver H. Buizer-Voskamp J. E. Dolan C. V. Boomsma D. I. (2016). Paternal age and psychiatric disorders: a review. *Am. J. Med. Genet. B Neuropsychiatr. Genet.* 174 202–213. 10.1002/ajmg.b.32508 27770494 PMC5412832

[B23] De Rubeis S. He X. Goldberg A. P. Poultney C. S. Samocha K. Ercument Cicek A. (2014). Synaptic, transcriptional and chromatin genes disrupted in autism. *Nature* 515 209–215. 10.1038/nature13772 25363760 PMC4402723

[B24] Dennis G. Sherman B. T. Hosack D. A. Yang J. Gao W. Lane H. (2003). DAVID: database for annotation, visualization, and integrated discovery. *Genome Biol.* 4:R60 10.1186/gb-2003-4-9-r60 PMC193660 12734009

[B25] Dong S. Walker M. F. Carriero N. J. DiCola M. Willsey A. J. Ye A. Y. (2014). De novo insertions and deletions of predominantly paternal origin are associated with autism spectrum disorder. *Cell Rep.* 9 16–23. 10.1016/j.celrep.2014.08.068 25284784 PMC4194132

[B26] Dou Y. Yang X. Li Z. Wang S. Zhang Z. Ye A. Y. (2017). Postzygotic single-nucleotide mosaicisms contribute to the etiology of autism spectrum disorder and autistic traits and the origin of mutations. *Hum. Mutat.* 38 1002–1013. 10.1002/humu.23255 28503910 PMC5518181

[B27] Durand C. M. Betancur C. Boeckers T. M. Bockmann J. Chaste P. Fauchereau F. (2006). Mutations in the gene encoding the synaptic scaffolding protein SHANK3 are associated with autism spectrum disorders. *Nat. Genet.* 39 25–27. 10.1038/ng1933 17173049 PMC2082049

[B28] Fombonne E. (2009). Epidemiology of pervasive developmental disorders. *Pediatr. Res.* 65 591–598. 10.1203/PDR.0b013e31819e7203 19218885

[B29] Frederiksen A. L. Duno M. Johnsen I. B. Nielsen M. F. Krøigård A. B. (2015). Asymptomatic parental mosaicism for osteogenesis imperfecta associated with a new splice site mutation in COL1A2. *Clin. Case Rep.* 4 972–978. 10.1902/jop.2010.090540.2 27761249 PMC5054473

[B30] Freed D. Pevsner J. (2016). The contribution of mosaic variants to autism spectrum disorder. *PLoS Genet.* 12:e1006245. 10.1371/journal.pgen.1006245 27632392 PMC5024993

[B31] Gaugler T. Klei L. Sanders S. J. Bodea C. A. Goldberg A. P. Lee A. B. (2014). Most genetic risk for autism resides with common variation. *Nat. Genet.* 46 881–885. 10.1038/ng.3039 25038753 PMC4137411

[B32] Geisheker M. R. Heymann G. Wang T. Coe B. P. Turner T. N. Stessman H. A. F. (2017). Hotspots of missense mutation identify neurodevelopmental disorder genes and functional domains. *Nat. Neurosci.* 20 1043–1051. 10.1038/nn.4589 28628100 PMC5539915

[B33] Genovese G. Kähler A. K. Handsaker R. E. Lindberg J. Rose S. A. Bakhoum S. F. (2014). Clonal hematopoiesis and blood-cancer risk inferred from blood DNA sequence. *N. Engl. J. Med.* 371 2477–2487. 10.1056/NEJMoa1409405 25426838 PMC4290021

[B34] Gilman S. R. Chang J. Xu B. Bawa T. S. Gogos J. A. Karayiorgou M. (2012). Diverse types of genetic variation converge on functional gene networks involved in schizophrenia. *Nat. Neurosci.* 15 1723–1728. 10.1038/nn.3261 23143521 PMC3689007

[B35] Gilman S. R. Iossifov I. Levy D. Ronemus M. Wigler M. Vitkup D. (2011). Rare de novo variants associated with autism implicate a large functional network of genes involved in formation and function of synapses. *Neuron* 70 898–907. 10.1016/j.neuron.2011.05.021 21658583 PMC3607702

[B36] Gratten J. Wray N. R. Peyrot W. J. McGrath J. J. Visscher P. M. Goddard M. E. (2016). Risk of psychiatric illness from advanced paternal age is not predominantly from de novo mutations. *Nat. Genet.* 48 718–724. 10.1038/ng.3577 27213288

[B37] Gronborg T. K. Schendel D. Parner E. T. (2013). Recurrence of autism spectrum disorders in full- and halfsiblings and trends over time: a population-based cohort study. *JAMA Pediatr.* 167 947–953. 10.1001/jamapediatrics.2013.2259 23959427 PMC4610344

[B38] Grove J. Ripke S. Als T. D. Mattheisen M. Bybjerg-grauholm J. Bækved-hansen M. (2017). Common risk variants identified in autism spectrum disorder. *bioRxiv* [Preprint] 10.1101/224774

[B39] Gupta S. Ellis S. E. Ashar F. N. Moes A. Bader J. S. Zhan J. (2014). Transcriptome analysis reveals dysregulation of innate immune response genes and neuronal activity-dependent genes in autism. *Nat. Commun.* 5:5748. 10.1038/ncomms6748 25494366 PMC4270294

[B40] He X. Sanders S. J. Liu L. De Rubeis S. Lim E. T. Sutcliffe J. S. (2013). Integrated model of de novo and inherited genetic variants yields greater power to identify risk genes. *PLoS Genet.* 9:e1003671. 10.1371/journal.pgen.1003671 23966865 PMC3744441

[B41] Hormozdiari F. Penn O. Borenstein E. Eichler E. E. (2015). The discovery of integrated gene networks for autism and related disorders. *Genome Res.* 25 142–154. 10.1101/gr.178855.114 25378250 PMC4317170

[B42] Huang D. Sherman B. Lempicki R. (2008). Systematic and integrative analysis of large gene lists using DAVID bioinformatics resources. *Nat. Protoc.* 4 44–57. 10.1038/nprot.2008.211 19131956

[B43] Huang D. W. Sherman B. T. Lempicki R. A. (2009). Bioinformatics enrichment tools: paths toward the comprehensive functional analysis of large gene lists. *Nucleic Acids Res.* 37 1–13. 10.1093/nar/gkn923 19033363 PMC2615629

[B44] Ionita-Laza I. Mccallum K. Xu B. Buxbaum J. D. (2016). A spectral approach integrating functional genomic annotations for coding and noncoding variants. *Nat. Genet.* 48 214–220. 10.1038/ng.3477 26727659 PMC4731313

[B45] Iossifov I. O’Roak B. J. Sanders S. J. Ronemus M. Krumm N. Levy D. (2014). The contribution of de novo coding mutations to autism spectrum disorder. *Nature* 515 216–221. 10.1038/nature13908 25363768 PMC4313871

[B46] Iossifov I. Ronemus M. Levy D. Wang Z. Hakker I. Rosenbaum J. (2012). De novo gene disruptions in children on the autistic spectrum. *Neuron* 74 285–299. 10.1016/j.neuron.2012.04.009 22542183 PMC3619976

[B47] Jakovcevski M. Akbarian S. (2013). Epigenetic mechanisms in neurodevelopmental and neurodegenerative disease. *Nat. Med.* 18 1194–1204. 10.1038/nm.2828.Epigenetic PMC3596876 22869198

[B48] Jamain S. Quach H. Betancur C. Råstam M. Colineaux C. Gillberg I. C. (2003). Mutations of the X-linked genes encoding neuroligins NLGN3 and NLGN4 are associated with autism. *Nat. Genet.* 34 27–29. 10.1038/ng1136 12669065 PMC1925054

[B49] Jamuar S. S. Lam A.-T. N. Kircher M. D’Gama A. M. Wang J. Barry B. J. (2014). Somatic mutations in cerebral cortical malformations. *N. Engl. J. Med.* 371 733–743. 10.1056/NEJMoa1314432 25140959 PMC4274952

[B50] Janecka M. Mill J. Basson M. A. Goriely A. Spiers H. Reichenberg A. (2017). Advanced paternal age effects in neurodevelopmental disorders—review of potential underlying mechanisms. *Transl. Psychiatry* 7:e1019. 10.1038/tp.2016.294 28140401 PMC5299396

[B51] Ji X. Kember R. L. Brown C. D. Bućan M. (2016). Increased burden of deleterious variants in essential genes in autism spectrum disorder. *Proc. Natl. Acad. Sci. U.S.A.* 113 15054–15059. 10.1073/pnas.1613195113 27956632 PMC5206557

[B52] Kang H. J. Kawasawa Y. I. Cheng F. Zhu Y. Xu X. Li M. (2011). Spatio-temporal transcriptome of the human brain. *Nature* 478 483–489. 10.1038/nature10523 22031440 PMC3566780

[B53] Kircher M. (2014). A general framework for estimating the relative pathogenicity of human genetic variants. *Nat. Genet.* 46 310–315. 10.1038/ng.2892.A 24487276 PMC3992975

[B54] Kong A. Frigge M. L. Masson G. Besenbacher S. Sulem P. Magnusson G. (2012). Rate of de novo mutations and the importance of father’s age to disease risk. *Nature* 488 471–475. 10.1038/nature11396 22914163 PMC3548427

[B55] Korb E. Herre M. Zucker-Scharff I. Gresack J. Allis C. D. Darnell R. B. (2017). Excess translation of epigenetic regulators contributes to fragile X syndrome and is alleviated by Brd4 inhibition. *Cell* 170 1209.e20–1223.e20. 10.1016/j.cell.2017.07.033 28823556 PMC5740873

[B56] Kosmicki J. A. Samocha K. E. Howrigan D. P. Sanders S. J. Slowikowski K. Lek M. (2017). Refining the role of de novo protein-truncating variants in neurodevelopmental disorders by using population reference samples. *Nat. Genet.* 49 504–510. 10.1038/ng.3789 28191890 PMC5496244

[B57] Krishnan A. Zhang R. Yao V. Theesfeld C. L. Wong A. K. Tadych A. (2016). Genome-wide prediction and functional characterization of the genetic basis of autism spectrum disorder. *Nat. Neurosci.* 19 1454–1462. 10.1038/nn.4353 27479844 PMC5803797

[B58] Krupp D. R. Barnard R. A. Duffourd Y. Evans S. A. Mulqueen R. M. Bernier R. (2017). Exonic mosaic mutations contribute risk for autism spectrum disorder. *Am. J. Hum. Genet.* 101 369–390. 10.1016/j.ajhg.2017.07.016 28867142 PMC5590950

[B59] Ku C. S. Vasiliou V. Cooper D. N. (2012). A new era in the discovery of de novo mutations underlying human genetic disease. *Hum. Genomics* 6:27. 10.1186/1479-7364-6-27 23232122 PMC3538533

[B60] Kumar P. Henikoff S. Ng P. C. (2009). Predicting the effects of coding non-synonymous variants on protein function using the SIFT algorithm. *Nat. Protoc.* 4 1073–1081. 10.1038/nprot.2009.86 19561590

[B61] Kurek K. C. Luks V. L. Ayturk U. M. Alomari A. I. Fishman S. J. Spencer S. A. (2012). Somatic mosaic activating mutations in *PIK3CA* cause CLOVES syn- drome. 90, 1108–111. *Am. J. Hum. Genet.* 90 1108–1115. 10.1016/j.ajhg.2012.05.006 22658544 PMC3370283

[B62] Lee J. H. Huynh M. Silhavy J. L. Kim S. Dixon-Salazar T. Heiberg A. (2012). De novo somatic mutations in components of the PI3K-AKT3-mTOR pathway cause hemimegalencephaly. *Nat. Genet.* 44 941–945. 10.1038/ng.2329 22729223 PMC4417942

[B63] Lehne B. Schlitt T. (2009). Protein-protein interaction databases: keeping up with growing interactomes. *Hum. Genomics* 3 291–297. 19403463 10.1186/1479-7364-3-3-291 PMC3500230

[B64] Leiserson M. Gramazio C. Hu J. Wu H. Laidlaw D. Raphael B. (2015). MAGI: visualization and collaborative annotation of genomic aberrations. *Nat. Methods* 12 483–484. 10.1038/nmeth.3412 26020500

[B65] Lek M. Karczewski K. J. Minikel E. V. Samocha K. E. Banks E. Fennell T. (2016). Analysis of protein-coding genetic variation in 60,706 humans. *Nature* 536 285–291. 10.1038/nature19057 27535533 PMC5018207

[B66] Lelieveld S. H. Spielmann M. Mundlos S. Veltman J. A. Gilissen C. (2015). Comparison of exome and genome sequencing technologies for the complete capture of protein-coding regions. *Hum. Mutat.* 36 815–822. 10.1002/humu.22813 25973577 PMC4755152

[B67] Levy D. Ronemus M. Yamrom B. Lee Y. H. Leotta A. Kendall J. (2011). Rare de novo and transmitted copy-number variation in autistic spectrum disorders. *Neuron* 70 886–897. 10.1016/j.neuron.2011.05.015 21658582

[B68] Lim E. T. Uddin M. De Rubeis S. Chan Y. Kamumbu A. S. Zhang X. (2017). Rates, distribution and implications of postzygotic mosaic mutations in autism spectrum disorder. *Nat. Neurosci.* 20 1217–1224. 10.1038/nn.4598 28714951 PMC5672813

[B69] Liu L. Lei J. Roeder K. (2015). Network assisted analysis to reveal the genetic basis of autism. *Ann. Appl. Stat.* 9 1571–1600. 10.1214/15-AOAS844 27134692 PMC4851445

[B70] Liu L. Lei J. Sanders S. Willsey A. (2014). DAWN: a framework to identify autism genes and subnetworks using gene expression and genetics. *Mol. Autism* 5 1–18. 10.1186/2040-2392-5-22 24602502 PMC4016412

[B71] Loomes R. Hull L. Mandy W. P. L. (2017). What is the male-to-female ratio in autism spectrum disorder? a systematic review and meta-analysis. *J. Am. Acad. Child Adolesc. Psychiatry* 56 466–474. 10.1016/j.jaac.2017.03.013 28545751

[B72] Ma D. Q. Salyakina D. Jaworski J. M. Konidari I. Patrice L. Andersen A. N. (2010). A genome-wide association study of autism reveals a common novel risk locus at 5p14.1. *Ann. Hum. Genet.* 73 263–273. 10.1111/j.1469-1809.2009.00523.x.A 19456320 PMC2918410

[B73] Marshall C. R. Noor A. Vincent J. B. Lionel A. C. Feuk L. Skaug J. (2008). Structural variation of chromosomes in autism spectrum disorder. *J. Hum. Genet.* 82 477–488. 10.1016/j.ajhg.2007.12.009 18252227 PMC2426913

[B74] McDowall M. D. Scott M. S. Barton G. J. (2009). PIPs: human protein-protein interaction prediction database. *Nucleic Acids Res.* 37 D651–D656. 10.1093/nar/gkn870 18988626 PMC2686497

[B75] McGrath J. J. Petersen L. Agerbo E. Mors O. Mortensen P. B. Pedersen C. B. (2014). A comprehensive assessment of parental age and psychiatric disorders. *JAMA Psychiatry* 71 301–309. 10.1001/jamapsychiatry.2013.4081 24452535

[B76] Merico D. Nicolson R. Patel R. V. Whitney J. Deflaux N. Bingham J. (2017). Whole genome sequencing resource identifies 18 new candidate genes for autism spectrum disorder. *Nat. Neurosci.* 20 602–611. 10.1038/nn.4524 28263302 PMC5501701

[B77] Michaelson J. J. Shi Y. Gujral M. Zheng H. Malhotra D. Jin X. (2012). Whole-genome sequencing in autism identifies hot spots for de novo germline mutation. *Cell* 151 1431–1442. 10.1016/j.cell.2012.11.019 23260136 PMC3712641

[B78] Mostafavi S. Ray D. Warde-Farley D. Grouios C. Morris Q. (2008). GeneMANIA: a real-time multiple association network integration algorithm for predicting gene function. *Genome Biol.* 9:S4. 10.1186/gb-2008-9-s1-s4 18613948 PMC2447538

[B79] Narzisi G. ORawe J. A. Iossifov I. Fang H. Lee Y.-H. Wang Z. (2014). Scalpel: accurate detection of de novo and transmitted INDELs within exome-capture data using micro-assembly. *Nat. Methods* 11 1033–1036. 10.1101/001370 25128977 PMC4180789

[B80] Neale B. M. Kou Y. Liu L. Ma’ayan A. Samocha K. E. Sabo A. (2012). Patterns and rates of exonic de novo mutations in autism spectrum disorders. *Nature* 485 242–245. 10.1038/nature11011 22495311 PMC3613847

[B81] Nguyen H. T. Bryois J. Kim A. Dobbyn A. Huckins L. M. Munoz-Manchado A. B. (2017). Integrated bayesian analysis of rare exonic variants to identify risk genes for schizophrenia and neurodevelopmental disorders. *Genome Med.* 9:114. 10.1186/s13073-017-0497-y 29262854 PMC5738153

[B82] O’Roak B. J. Deriziotis P. Lee C. Vives L. Schwartz J. J. Girirajan S. (2011). Exome sequencing in sporadic autism spectrum disorders identifies severe de novo mutations. *Nat. Genet.* 43 585–589. 10.1038/ng.835 21572417 PMC3115696

[B83] O’Roak B. J. Vives L. Girirajan S. Karakoc E. Krumm N. Coe B. P. (2012). Sporadic autism exomes reveal a highly interconnected protein network of de novo mutations. *Nature* 485 246–250. 10.1038/nature10989 22495309 PMC3350576

[B84] Ozonoff S. Young G. S. Carter A. Messinger D. Yirmiya N. Zwaigenbaum L. (2011). Recurrence risk for autism spectrum disorders: a baby siblings research consortium study. *Pediatrics* 128 e488–e495. 10.1542/peds.2010-2825 21844053 PMC3164092

[B85] Pagnamenta A. T. Lise S. Harrison V. Stewart H. Jayawant S. Quaghebeur G. (2012). Exome sequencing can detect pathogenic mosaic mutations present at low allele frequencies. *J. Hum. Genet.* 57 70–72. 10.1038/jhg.2011.128 22129557

[B86] Parikshak N. N. Gandal M. J. Geschwind D. H. (2015). Systems biology and gene networks in neurodevelopmental and neurodegenerative disorders. *Nat. Rev.* 16 441–458. 10.1038/nrg3934 26149713 PMC4699316

[B87] Parikshak N. N. Luo R. Zhang A. Won H. Lowe J. K. Chandran V. (2013). Integrative functional genomic analyses implicate specific molecular pathways and circuits in autism. *Cell* 155 1008–1021. 10.1016/j.cell.2013.10.031 24267887 PMC3934107

[B88] Parikshak N. N. Swarup V. Belgard T. G. Irimia M. Ramaswami G. Gandal M. J. (2016). Genome-wide changes in lncRNA, splicing, and regional gene expression patterns in autism. *Nature* 540 423–427. 10.1038/nature20612 27919067 PMC7102905

[B89] Patel Z. H. Kottyan L. C. Lazaro S. Williams M. S. Ledbetter D. H. Tromp G. (2014). The struggle to find reliable results in exome sequencing data: filtering out Mendelian errors. *Front. Genet.* 5:16. 10.3389/fgene.2014.00016 24575121 PMC3921572

[B90] Petrovski S. Wang Q. Heinzen E. L. Allen A. S. Goldstein D. B. (2013). Genic intolerance to functional variation and the interpretation of personal genomes. *PLoS Genet.* 9:e1003709. 10.1371/journal.pgen.1003709 23990802 PMC3749936

[B91] Pieras J. I. Muñoz-cabello B. Borrego S. Marcos I. Sanchez J. Madruga M. (2012). Somatic mosaicism for Y120X mutation in the *MECP2* gene causes atypical Rett syndrome in a male. *Brain Dev.* 33 608–611. 10.1016/j.braindev.2010.09.012 20970936

[B92] Pinto D. Delaby E. Merico D. Barbosa M. Merikangas A. Klei L. (2014). Convergence of genes and cellular pathways dysregulated in autism spectrum disorders. *Am. J. Hum. Genet.* 94 677–694. 10.1016/j.ajhg.2014.03.018 24768552 PMC4067558

[B93] Pinto D. Pagnamenta A. T. Klei L. Anney R. Merico D. Regan R. (2010). Functional impact of global rare copy number variation in autism spectrum disorder. *Nature* 466 368–372. 10.1038/nature09146.Functional 20531469 PMC3021798

[B94] Pisansky M. T. Young A. E. O’Connor M. B. Gottesman I. I. Bagchi A. Gewirtz J. C. (2017). Mice lacking the chromodomain helicase DNA-binding 5 chromatin remodeler display autism-like characteristics. *Transl. Psychiatry* 7:e1152. 10.1038/tp.2017.111 28608855 PMC5537637

[B95] Platt R. J. Zhou Y. Slaymaker I. M. Shetty A. S. Weisbach N. R. Kim J.-A. (2017). Chd8 mutation leads to autistic-like behaviors and impaired striatal circuits. *Cell Rep.* 19 335–350. 10.1016/j.celrep.2017.03.052 28402856 PMC5455342

[B96] Poduri A. Evrony G. D. Cai X. Walsh C. A. (2013). Somatic mutation, genomic variation, and neurological disease. *Science* 341:1237758. 10.1126/science.1237758 23828942 PMC3909954

[B97] Polimanti R. Gelernter J. (2017). Widespread signatures of positive selection in common risk alleles associated to autism spectrum disorder. *PLoS Genet.* 13:e1006618. 10.1371/journal.pgen.1006618 28187187 PMC5328401

[B98] Poultney C. S. Goldberg A. P. Drapeau E. Kou Y. Harony-Nicolas H. Kajiwara Y. (2013). Identification of small exonic CNV from whole-exome sequence data and application to autism spectrum disorder. *Am. J. Hum. Genet.* 93 607–619. 10.1016/j.ajhg.2013.09.001 24094742 PMC3791269

[B99] Risch N. Hoffmann T. J. Anderson M. Croen L. A. Grether J. K. Windham G. C. (2014). Familial recurrence of autism spectrum disorder: evaluating genetic and environmental contributions. *Am. J. Psychiatry* 171 1206–1213. 10.1176/appi.ajp.2014.13101359 24969362

[B100] Robinson E. B. Samocha K. E. Kosmicki J. A. McGrath L. Neale B. M. Perlis R. H. (2014). Autism spectrum disorder severity reflects the average contribution of de novo and familial influences. *Proc. Natl. Acad. Sci. U.S.A.* 111 15161–15165. 10.1073/pnas.1409204111 25288738 PMC4210299

[B101] Roelfsema J. White S. Ariyürek Y. Barthold D. Niedrist D. Papadia F. (2005). Genetic heterogeneity in Rubinstein–Taybi syndrome: mutations in both the CBP and EP300 genes cause disease. *Am. J. Hum. Genet.* 76 576–580. 10.1086/429130 15706485 PMC1199295

[B102] Ronan J. L. Wu W. Crabtree G. R. (2013). From neural development to cognition: unexpected roles for chromatin. *Nat. Rev. Genet.* 14 347–359. 10.1038/nrg3413 23568486 PMC4010428

[B103] Ronemus M. Iossifov I. Levy D. Wigler M. (2014). The role of de novo mutations in the genetics of autism spectrum disorders. *Nat. Rev. Genet.* 15 133–141. 10.1038/nrg3585 24430941

[B104] Rossin E. J. Lage K. Raychaudhuri S. Xavier R. J. Tatar D. Benita Y. (2011). Proteins encoded in genomic regions associated with immune-mediated disease physically interact and suggest underlying biology. *PLoS Genet.* 7:e1001273. 10.1371/journal.pgen.1001273 21249183 PMC3020935

[B105] Samocha K. E. Kosmicki J. A. Karczewski K. J. O’Donnell-Luria A. H. Pierce-Hoffman E. MacArthur D. G. (2017). Regional missense constraint improves variant deleteriousness prediction. *bioRxiv* [Preprint] 10.1101/148353

[B106] Sanders S. J. Ercan-Sencicek A. G. Hus V. Luo R. Murtha M. T. Moreno-De-Luca D. (2011). Multiple recurrent de novo CNVs, including duplications of the 7q11.23 williams syndrome region, are strongly associated with autism. *Neuron* 70 863–885. 10.1016/j.neuron.2011.05.002 21658581 PMC3939065

[B107] Sanders S. J. He X. Willsey A. J. Ercan-Sencicek A. G. Samocha K. E. Cicek A. E. (2015). Insights into autism spectrum disorder genomic architecture and biology from 71 risk loci. *Neuron* 87 1215–1233. 10.1016/j.neuron.2015.09.016 26402605 PMC4624267

[B108] Sanders S. J. Murtha M. T. Gupta A. R. Murdoch J. D. Raubeson M. J. Willsey A. J. (2012). De novo mutations revealed by whole-exome sequencing are strongly associated with autism. *Nature* 485 237–241. 10.1038/nature10945 22495306 PMC3667984

[B109] Sandin S. Lichtenstein P. Kuja-Halkola R. Hultman C. Larsson H. Reichenberg A. (2017). The heritability of autism spectrum disorder analysis method B. *JAMA* 318 1182–1184. 10.1001/jama.2017.12141 28973605 PMC5818813

[B110] Sebat J. Lakshmi B. Malhotra D. Troge J. Lese-C. Walsh T. (2010). Strong association of de novo copy number mutations with autism. *Science* 316 445–449. 10.1126/science.1138659.Strong PMC2993504 17363630

[B111] Sener E. F. Canatan H. Ozkul Y. (2016). Recent advances in autism spectrum disorders: applications of whole exome sequencing technology. *Psychiatry Investig.* 13 255–264. 10.4306/pi.2016.13.3.255 27247591 PMC4878959

[B112] Shohat S. Ben-David E. Shifman S. (2017). Varying intolerance of gene pathways to mutational classes explain genetic convergence across neuropsychiatric disorders. *Cell Rep.* 18 2217–2227. 10.1016/j.celrep.2017.02.007 28249166

[B113] Short P. J. McRae J. F. Gallone G. Sifrim A. Won H. Geschwind D. H. (2018). De novo mutations in regulatory elements in neurodevelopmental disorders. *Nature* 555 611–616. 10.1038/nature25983 29562236 PMC5912909

[B114] Song L. Langfelder P. Horvath S. Eisena M. Spellman P. Brown P. (2012). Comparison of co-expression measures: mutual information, correlation, and model based indices. *BMC Bioinformatics* 13:328. 10.1186/1471-2105-13-328 23217028 PMC3586947

[B115] Steffeneburg S. (1989). A twin study of autism in Denmark, Finland, Iceland, Norway, and Sweden. *J. Child Psychol. Psychiatry* 30 405–416. 10.1111/j.1469-7610.1989.tb00254.x 2745591

[B116] Stein J. L. Parikshak N. N. Geschwind D. H. (2013). Rare inherited variation in autism: beginning to see the forest and a few trees. *Neuron* 77 209–211. 10.1016/j.neuron.2013.01.010 23352155 PMC3691080

[B117] Tsurusaki I. Okamoto N. Ohashi H. Kosho T. Imai Y. Hibi-Ko Y. (2012). Mutations affecting components of the SWI/SNF complex cause coffin–siris syndrome. *Nat. Genet.* 44 376–378. 10.1038/ng.2219 22426308

[B118] Turner T. N. Coe B. P. Dickel D. E. Hoekzema K. Nelson B. J. Zody M. C. (2017). Genomic patterns of de novo mutation in simplex autism. *Cell* 171 710.e12–722.e12. 10.1016/j.cell.2017.08.047 28965761 PMC5679715

[B119] Uddin M. Tammimies K. Pellecchia G. Alipanahi B. Hu P. Wang Z. (2014). Brain-expressed exons under purifying selection are enriched for *de novo* mutations in autism spectrum disorder. *Nat. Genet.* 46 742–747. 10.1038/ng.2980 24859339

[B120] Uetz P. Fields S. Giot L. Cagney G. Mansfield T. A. Judson R. S. (2000). A comprehensive analysis of protein-protein interactions in *Saccharomyces cerevisiae*. *Nature* 403 623–627. 10.1038/35001009 10688190

[B121] van Bokhoven H. (2011). Genetic and epigenetic networks in intellectual disabilities. *Annu. Rev. Genet.* 45 81–104. 10.1146/annurev-genet-110410-132512 21910631

[B122] van Dam S. Võsa U. van der Graaf A. Franke L. de Magalhães J. P. (2017). Gene co-expression analysis for functional classification and gene–disease predictions. *Brief. Bioinform.* 19 575–592. 10.1093/bib/bbw139 28077403 PMC6054162

[B123] Van Houdt J. K. Nowakowska B. A. Sousa S. B. van Schaik B. D. Seuntjens E. Avonce N. (2012). Heterozygous missense mutations in SMARCA2 cause nicolaides–baraitser syndrome. *Nat. Genet.* 44 445–449. 10.1038/ng.1105 22366787

[B124] Vissers L. Van Ravenswaaij C. Admiraal R. Hurst J. De Vries B. Janssen I. (2004). Mutations in a new member of the chromodomain gene family cause charge syndrome. *Nat. Genet.* 36 955–957. 10.1038/ng1407 15300250

[B125] Voineagu I. Wang X. Johnston P. Lowe J. K. Tian Y. Horvath S. (2011). Transcriptomic analysis of autistic brain reveals convergent molecular pathology. *Nature* 474 380–384. 10.1038/nature10110 21614001 PMC3607626

[B126] Warr A. Robert C. Hume D. Archibald A. Deeb N. Watson M. (2015). Exome sequencing: current and future perspectives. *G* 3 5 1543–1550. 10.1534/g3.115.018564 26139844 PMC4528311

[B127] Weiss L. A. Arking D. E. Daly M. J. Chakravarti A. Arking D. E. Brune C. W. (2009). A genome-wide linkage and association scan reveals novel loci for autism. *Nature* 461 802–808. 10.1038/nature08490 19812673 PMC2772655

[B128] Wen Y. Alshikho M. J. Herbert M. R. (2016). Pathway network analyses for autism reveal multisystem involvement, major overlaps with other diseases and convergence upon MAPK and calcium signaling. *PLoS One* 11:e0153329. 10.1371/journal.pone.0153329 27055244 PMC4824422

[B129] Werling D. M. Brand H. An J. Stone M. R. Zhu L. Glessner J. T. (2018). An analytical framework for whole genome sequence association studies and its implications for autism spectrum disorder. *Nat. Genet.* 50 727–736. 10.1038/s41588-018-0107-y 29700473 PMC5961723

[B130] Willsey A. J. Sanders S. J. Li M. Dong S. Tebbenkamp A. T. Muhle R. A. (2013). Coexpression networks implicate human midfetal deep cortical projection neurons in the pathogenesis of autism. *Cell* 155 997–1007. 10.1016/j.cell.2013.10.020 24267886 PMC3995413

