# Supplementary material for: De novo Mutations (DNMs) in Autism Spectrum Disorder (ASD): Pathway and Network Analysis
Source: Front Genet. 2018 Sep 21;9:406. doi: 10.3389/fgene.2018.00406 (PMC6160549; doi:10.3389/fgene.2018.00406)
Supplement: Supplementary file 1 [file Table_1.doc]

Supplementary Material

De novo mutations (DNMs) in Autism Spectrum Disorder (ASD): pathway and network analysis

Aitana Alonso-González^1^,, Cristina Rodriguez-Fontenla^2 a^ & Angel Carracedo*^1,2 a^

*** Correspondence: Cristina Rodriguez-Fontenla**: [m](mailto:angel.carracedo@usc.es)ariacristina.rodriguez.fontenla@usc.es

# Supplementary Tables

**Supplementary Table 1** High confidence (score 1), Strong candidate (score 2), and syndromic (s) genes according to Sfari Gene (https://gene.sfari.org/) criteria are represented in this table. Molecular function following GO terms, OMIM phenotypes and main studies reporting those genes associated in ASD supplement the information available in Sfari Gene.

| **Gene** | **Sfari Score** | **Chromosome band** | **Go ontologies: Molecular function** | **OMIM** | **References** |
| --- | --- | --- | --- | --- | --- |
| ***ANK2*** | 1 |  | structural constituent of cytoskeleton; protein binding; enzyme binding; protein kinase binding; spectrin binding; protein binding, bridging; ion channel binding; ATPase binding | Cardiac arrhythmia, ankyrin-B-related (600919), Long QT syndrome 4 (600919) | (De Rubeis et al., 2014; Iossifov et al., 2012, 2014; Krumm et al., 2015; Merico et al., 2017; Willsey et al., 2013) |
| ***ASH1L*** | 1 | 1q22 | transferase activity; methyltransferase activity; metal ion binding; histone-lysine N-methyltransferase activity; histone methyltransferase activity (H3-K4 specific); histone methyltransferase activity (H3-K36 specific); DNA binding; chromatin binding | Mental retardation, autosomal dominant 52 (617796) | (De Rubeis et al., 2014; Iossifov et al., 2014; Merico et al., 2017; Willsey et al., 2013) |
| ***CUL3*** | 1 | 2q36.2 | ubiquitin-protein transferase activity; ubiquitin protein ligase binding; ubiquitin protein ligase activity; protein homodimerization activity; protein heterodimerization activity; protein binding; POZ domain binding; Notch binding; cyclin binding | Pseudohypoaldosteronism, type IIE (614496) | (De Rubeis et al., 2014; Merico et al., 2017; O’Roak et al., 2012b) |
| ***DSCAM*** | 1 | 21q22.2 | protein binding |  | (Iossifov et al., 2014; Merico et al., 2017) |
| ***GRIN2B*** | 1 | 12p13.1 | zinc ion binding; receptor activity; Ras guanyl-nucleotide exchange factor activity; protein binding; NMDA glutamate receptor activity; metal ion binding; ionotropic glutamate receptor activity; ion channel activity; glycine binding; glutamate-gated calcium ion channel activity; glutamate binding; extracellular-glutamate-gated ion channel activity | Epileptic encephalopathy, early infantile, 27 (616139), Mental retardation, autosomal dominant 6 (613970) | (De Rubeis et al., 2014; Iossifov et al., 2012, 2014; Lim et al., 2017; O’Roak et al., 2012b) |
| ***KATNAL2*** | 1 | 18q21.1 | protein binding; nucleotide binding; microtubule-severing ATPase activity; hydrolase activity; ATP binding | {Autism susceptibility 1} (209850) | (De Rubeis et al., 2014; Merico et al., 2017; O’Roak et al., 2012b; Sanders et al., 2012) |
| ***KMT5B*** | 1 | 11q13.2 | transferase activity; methyltransferase activity; histone-lysine N-methyltransferase activity; histone methyltransferase activity (H4-K20 specific) | Mental retardation, autosomal dominant 51 (617788) | (De Rubeis et al., 2014; Iossifov et al., 2012; Merico et al., 2017; Sanders et al., 2012) |
| ***MYT1L*** | 1 | 2p25.3 | zinc ion binding; transcriptional repressor activity, RNA polymerase II core promoter proximal region sequence-specific binding; transcription factor activity, sequence-specific DNA binding; metal ion binding; DNA binding | Mental retardation, autosomal dominant 39 (616521) | (De Rubeis et al., 2014; Iossifov et al., 2014; Merico et al., 2017) |
| ***NAA15*** | 1 | 4q31.1 | RNA binding; ribosome binding; protein binding; peptide alpha-N-acetyltransferase activity; acetyltransferase activity | Mental retardation, autosomal dominant 50 (617787) | (De Rubeis et al., 2014; Merico et al., 2017) |
| ***RELN*** | 1 | 7q22.1 | very-low-density lipoprotein particle receptor binding; serine-type peptidase activity; protein serine/threonine/tyrosine kinase activity; peptidase activity; metal ion binding; lipoprotein particle receptor binding; hydrolase activity | Lissencephaly 2 (Norman-Roberts type) (257320), {Epilepsy, familial temporal lobe, 7} (616436) | (De Rubeis et al., 2014; Iossifov et al., 2012; Merico et al., 2017) |
| ***SCN2A*** | 1 | 2q24.3 | voltage-gat(Krupp et al., 2017)ed sodium channel activity; voltage-gated ion channel activity; sodium channel activity; ion channel activity; | Epileptic encephalopathy, early infantile, 11 (613721), Seizures, benign familial infantile, 3 (607745) | (De Rubeis et al., 2014; Iossifov et al., 2012, 2014; Krumm et al., 2015; Krupp et al., 2017; Lim et al., 2017; Merico et al., 2017; Sanders et al., 2012; Weiss et al., 2009) |
| ***TBR1*** | 1 | 2q24.2 | transcription factor activity, sequence-specific DNA binding; RNA polymerase II core promoter sequence-specific DNA binding; protein kinase binding; DNA binding |  | (De Rubeis et al., 2014; Neale et al., 2012; O’Roak et al., 2012b) |
| ***BCKDK*** | 2 | 16p11.2 | transferase activity, transferring phosphorus-containing groups; transferase activity; protein serine/threonine kinase activity; protein kinase activity; protein binding; nucleotide binding; kinase activity; ATP binding; [3-methyl-2-oxobutanoate dehydrogenase (acetyl-transferring)] kinase activity | Branched-chain ketoacid dehydrogenase kinase deficiency (614923) | (Novarino et al., 2012) |
| ***CACNA1D*** | 2 | 3p21.1 | voltage-gated ion channel activity; voltage-gated calcium channel activity involved SA node cell action potential; voltage-gated calcium channel activity involved in cardiac muscle cell action potential; voltage-gated calcium channel activity; voltage-gated calcium channel activity; metal ion binding; ion channel activity; high voltage-gated calcium channel activity; calcium channel activity; ankyrin binding; alpha-actinin binding | Primary aldosteronism, seizures, and neurologic abnormalities (615474), Sinoatrial node dysfunction and deafness (614896) | (De Rubeis et al., 2014; Iossifov et al., 2012; Lim et al., 2017; O’Roak et al., 2012b) |
| ***CACNA1H*** | 2 | 16p13.3 | voltage-gated sodium channel activity; voltage-gated ion channel activity; voltage-gated calcium channel activity; scaffold protein binding; metal ion binding; low voltage-gated calcium channel activity; ion channel activity; calcium channel activity | Hyperaldosteronism, familial, type IV (617027), {Epilepsy, childhood absence, susceptibility to, 6} (611942), {Epilepsy, idiopathic generalized, susceptibility to, 6} (611942) | (Iossifov et al., 2014) |
| ***CACNA2D3*** | 2 | 3p21.1-p14.3 | voltage-gated ion channel activity; metal ion binding; calcium channel activity |  | (De Rubeis et al., 2014; Iossifov et al., 2012; Merico et al., 2017) |
| ***CNTN4*** | 2 | 3p26.3-p26.2 |  |  | (Lim et al., 2017) |
| ***CTNND2*** | 2 | 5p15.2 | protein binding; beta-catenin binding |  | (Turner et al., 2015) |
| ***DIP2C*** | 2 | 10p15.3 | molecular_function; catalytic activity |  | (Iossifov et al., 2014; Merico et al., 2017) |
| ***ERBIN*** | 2 | 5q12.3 | structural constituent of cytoskeleton; receptor binding; protein binding; integrin binding; ErbB-2 class receptor binding |  | (De Rubeis et al., 2014; Krumm et al., 2015; Sanders et al., 2015) |
| ***GABRB3*** | 2 | 15q12 | ion channel activity; identical protein binding; GABA-gated chloride ion channel activity; GABA-A receptor activity; extracellular ligand-gated ion channel activity; chloride channel activity | Epileptic encephalopathy, early infantile, 43 (617113), {Epilepsy, childhood absence, susceptibility to, 5} (612269) | (De Rubeis et al., 2014; Iossifov et al., 2012; Krumm et al., 2015) |
| ***GIGYF2*** | 2 | 2q37.1 | RNA binding; protein binding; proline-rich region binding; cadherin binding | {Parkinson disease 11} (607688) | (De Rubeis et al., 2014; Iossifov et al., 2014; Krumm et al., 2015; Lim et al., 2017) |
| ***GRIA1*** | 2 | 5q33.2 | transmitter-gated ion channel activity involved in regulation of postsynaptic membrane potential; receptor activity; protein binding; PDZ domain binding; neurotransmitter receptor activity involved in regulation of postsynaptic cytosolic calcium ion concentration; ionotropic glutamate receptor activity; ion channel activity; glutamate receptor activity; extracellular-glutamate-gated ion channel activity; excitatory extracellular ligand-gated ion channel activity; amyloid-beta binding; AMPA glutamate receptor activity |  | (De Rubeis et al., 2014; Iossifov et al., 2014) |
| ***GRIP1*** | 2 | 12q14.3 | transcription coactivator activity; receptor signaling complex scaffold activity; protein C-terminus binding; protein binding; glucocorticoid receptor binding; beta-catenin binding; androgen receptor binding | Fraser syndrome 3 (617667) | (Kenny et al., 2014) |
| ***ILF2*** | 2 | 1q21.3 | DNA binding; RNA binding; double-stranded RNA binding; protein binding; ATP binding; transferase activity |  | (De Rubeis et al., 2014; Iossifov et al., 2014; Sanders et al., 2015) |
| ***INTS6*** | 2 | 13q14.3 | transmembrane signaling receptor activity; protein binding |  | (Krumm et al., 2015; Sanders et al., 2015) |
| ***IRF2BPL*** | 2 | 14q24.3 | molecular_function; metal ion binding |  | (Iossifov et al., 2014; Sanders et al., 2015) |
| ***KAT2B*** | 2 | 3p24.3 | transferase activity, transferring acyl groups; transferase activity; transcription factor binding; transcription cofactor activity; transcription coactivator activity; RNA polymerase II regulatory region sequence-specific DNA binding; protein kinase binding; protein complex binding; protein binding; N-acetyltransferase activity; lysine N-acetyltransferase activity, acting on acetyl phosphate as donor; histone deacetylase binding; histone acetyltransferase activity; cyclin-dependent protein serine/threonine kinase inhibitor activity; chromatin binding; acetyltransferase activity |  | (Iossifov et al., 2014; Pinto et al., 2014; Sanders et al., 2015) |
| ***KDM5B*** | 2 | 1q32.1 | DNA binding; transcription factor activity, sequence-specific DNA binding; transcription corepressor activity; protein binding; zinc ion binding; oxidoreductase activity; histone demethylase activity; histone demethylase activity (H3-K4 specific); histone demethylase activity (H3-trimethyl-K4 specific); histone demethylase activity (H3-dimethyl-K4 specific); histone binding; metal ion binding; dioxygenase activity; sequence-specific double-stranded DNA binding |  | (De Rubeis et al., 2014; Iossifov et al., 2014; Krumm et al., 2015; Merico et al., 2017) |
| ***KDM6A*** | 2 | Xp11.3 | RNA polymerase II core promoter proximal region sequence-specific DNA binding; oxidoreductase activity; metal ion binding; identical protein binding; histone demethylase activity (H3-K27 specific); histone demethylase activity; dioxygenase activity; chromatin DNA binding | Kabuki syndrome 2 (300867) | (Merico et al., 2017) |
| ***KMT2C*** | 2 | 7q36.1 | transferase activity, transferring acyl groups; transferase activity; RNA binding; protein binding; methyltransferase activity; metal ion binding; histone-lysine N-methyltransferase activity; histone methyltransferase activity (H3-K4 specific); DNA binding | Kleefstra syndrome 2 (617768) | (De Rubeis et al., 2014; Iossifov et al., 2014; Krumm et al., 2015; Krupp et al., 2017; Lim et al., 2017; Merico et al., 2017; O’Roak et al., 2012b) |
| ***MED13*** | 2 | 17q23.2 | vitamin D receptor binding; transcription cofactor activity; transcription coactivator activity; thyroid hormone receptor binding; RNA polymerase II transcription cofactor activity; receptor activity; ligand-dependent nuclear receptor transcription coactivator activity |  | (Iossifov et al., 2014; Merico et al., 2017) |
| ***MED13L*** | 2 | 12q24.21 | RNA polymerase II transcription cofactor activity | Mental retardation and distinctive facial features with or without cardiac defects (616789), Transposition of the great arteries, dextro-looped 1 (608808) | (Iossifov et al., 2012, 2014) |
| ***MET*** | 2 | 7q31.2 | transmembrane receptor protein tyrosine kinase activity; transferase activity; Ras guanyl-nucleotide exchange factor activity; protein tyrosine kinase activity; protein phosphatase binding; protein kinase activity; protein binding; phosphatidylinositol-4,5-bisphosphate 3-kinase activity; nucleotide binding; kinase activity; identical protein binding; hepatocyte growth factor-activated receptor activity; ATP binding | ?Deafness, autosomal recessive 97 (616705), Hepatocellular carcinoma, childhood type, somatic (114550), Renal cell carcinoma, papillary, 1, familial and somatic (605074), {Osteofibrous dysplasia, susceptibility to} (607278) | (Lim et al., 2017) |
| ***MSNP1AS*** | 2 |  |  |  | (Kerin et al., 2012) |
| ***NCKAP1*** | 2 | 2q32.1 | Rac GTPase binding; protein complex binding; protein binding |  | (De Rubeis et al., 2014; Iossifov et al., 2012, 2014) |
| ***NLGN3*** | 2 | Xq13.1 | scaffold protein binding; receptor activity; protein binding; neurexin family protein binding; cell adhesion molecule binding; carboxylic ester hydrolase activity | {Asperger syndrome susceptibility, X-linked 1} (300494), {Autism susceptibility, X-linked 1} (300425) | (Iossifov et al., 2014; Merico et al., 2017; Sanders et al., 2015) |
| ***NRXN1*** | 2 | 2p16.3 | type 1 fibroblast growth factor receptor binding; transmembrane signaling receptor activity; receptor binding; receptor activity; protein binding; neuroligin family protein binding; metal ion binding; cell adhesion molecule binding; calcium-dependent protein binding; calcium ion binding; calcium channel regulator activity; acetylcholine receptor binding | Pitt-Hopkins-like syndrome 2 (614325), {Schizophrenia, susceptibility to, 17} (614332) | (Brandler et al., 2016; De Rubeis et al., 2014; Iossifov et al., 2012) |
| ***PHF3*** | 2 | 6q12 | molecular_function; metal ion binding |  | (Iossifov et al., 2014; Merico et al., 2017) |
| ***PTCHD1*** | 2 | Xp22.11 | molecular_function | {Autism, susceptibility to, X-linked 4} (300830) | (Pinto et al., 2014) |
| ***RANBP17*** | 2 | 5q35.1 | Ran GTPase binding; nuclear export signal receptor activity; GTP binding |  | (De Rubeis et al., 2014; Iossifov et al., 2014; Sanders et al., 2015) |
| ***RIMS1*** | 2 | 6q13 | RNA binding; Rab GTPase binding; protein binding; metal ion binding; ion channel binding; GTPase regulator activity | Cone-rod dystrophy 7 (603649) | (Iossifov et al., 2012) |
| ***SCN9A*** | 2 | 2q24.3 | ion channel activity; voltage-gated ion channel activity; voltage-gated sodium channel activity; sodium channel activity; sodium ion binding | Epilepsy, generalized, with febrile seizures plus, type 7 (613863), Erythermalgia, primary (133020), Febrile seizures, familial, 3B (613863), HSAN2D, autosomal recessive (243000), Insensitivity to pain, congenital (243000), Paroxysmal extreme pain disorder (167400), Small fiber neuropathy (133020), {Dravet syndrome, modifier of} (607208) | (Rubinstein et al., 2018) |
| ***SHANK2*** | 2 | 11q13.3-q13.4 | SH3 domain binding; protein binding; ionotropic glutamate receptor binding; GKAP/Homer scaffold activity | {Autism susceptibility 17} (613436) | (Merico et al., 2017; Sanders et al., 2012) |
| ***SLC6A1*** | 2 | 3p25.3 | neurotransmitter:sodium symporter activity; gamma-aminobutyric acid:sodium symporter activity; symporter activity; metal ion binding | Myoclonic-atonic epilepsy (616421) | (De Rubeis et al., 2014; Iossifov et al., 2014; Krumm et al., 2015; Merico et al., 2017; Sanders et al., 2012, 2015) |
| ***SMARCC2*** | 2 | 12q13.2 | transcription coactivator activity; RNA polymerase II distal enhancer sequence-specific DNA binding; RNA polymerase II core promoter proximal region sequence-specific DNA binding; protein binding; nucleosomal DNA binding; DNA binding; chromatin binding |  | (Iossifov et al., 2012; Merico et al., 2017; Neale et al., 2012) |
| ***SPAST*** | 2 | 2p22.3 | nucleotide binding; catalytic activity; protein binding; ATP binding; microtubule binding; microtubule-severing ATPase activity; hydrolase activity; protein complex binding; alpha-tubulin binding; beta-tubulin binding | Spastic paraplegia 4, autosomal dominant (182601) | (Iossifov et al., 2014; Merico et al., 2017; Neale et al., 2012; Sanders et al., 2015) |
| ***SRCAP*** | 2 | 16p11.2 | transcription coactivator activity; protein binding; nucleotide binding; hydrolase activity; histone acetyltransferase activity; helicase activity; DNA binding; ATP binding | Floating-Harbor syndrome (136140) | (De Rubeis et al., 2014; Iossifov et al., 2014; Merico et al., 2017) |
| ***SRSF11*** | 2 | 1p31.1 | RNA binding; protein binding; nucleic acid binding |  | (De Rubeis et al., 2014; Lim et al., 2017; Merico et al., 2017) |
| ***TBL1XR1*** | 2 | 3q26.32 | transcription regulatory region DNA binding; transcription corepressor activity; protein N-terminus binding; protein binding; histone binding; DNA binding; beta-catenin binding | Mental retardation, autosomal dominant 41 (616944), Pierpont syndrome (602342) | (O’Roak et al., 2012b) |
| ***TCF20*** | 2 | 22q13.2 | transcription regulatory region DNA binding; transcription factor activity, sequence-specific DNA binding; transcription coactivator activity; RNA binding; metal ion binding; DNA binding |  | (Merico et al., 2017) |
| ***TNRC6B*** | 2 | 22q13.1 | RNA binding; protein binding; nucleic acid binding |  | (Dong et al., 2014; Iossifov et al., 2014) |
| ***UBN2*** | 2 | 7q34 | unknown |  | (De Rubeis et al., 2014; Iossifov et al., 2014; Merico et al., 2017) |
| ***USP7*** | 2 | 16p13.2 | ubiquitin protein ligase binding; transcription factor binding; thiol-dependent ubiquitinyl hydrolase activity; thiol-dependent ubiquitin-specific protease activity; protein C-terminus binding; protein binding; peptidase activity; p53 binding; hydrolase activity; cysteine-type peptidase activity; cysteine-type endopeptidase activity |  | (De Rubeis et al., 2014; Iossifov et al., 2014) |
| ***WDFY3*** | 2 | 4q21.23 | protein binding; metal ion binding; lipid binding; beta-N-acetylglucosaminylglycopeptide beta-1,4-galactosyltransferase activity; 1-phosphatidylinositol binding | ?Microcephaly 18, primary, autosomal dominant (617520) | (De Rubeis et al., 2014; Iossifov et al., 2012, 2014; Merico et al., 2017) |
| ***ADNP*** | s1 | 20q13.13 | protein binding; peptide binding; nucleic acid binding; metal ion binding; DNA binding; copper ion binding; chromatin binding; beta-tubulin binding | Helsmoortel-van der Aa syndrome (615873) | (De Rubeis et al., 2014; Merico et al., 2017; O’Roak et al., 2012b) |
| ***ARID1B*** | s1 | 6q25.3 | transcription coactivator activity; protein binding; DNA binding | Coffin-Siris syndrome 1 (135900) | (De Rubeis et al., 2014; Krumm et al., 2015; Lim et al., 2017; Merico et al., 2017; O’Roak et al., 2012b) |
| ***ASXL3*** | s1 | 18q12.1 | metal ion binding; DNA binding | Bainbridge-Ropers syndrome (615485) | (De Rubeis et al., 2014; Merico et al., 2017) |
| ***CHD8*** | s1 | 14q11.2 | protein binding; p53 binding; nucleotide binding; methylated histone binding; hydrolase activity, acting on acid anhydrides; hydrolase activity; histone binding; helicase activity; DNA-dependent ATPase activity; DNA helicase activity; DNA binding; chromatin binding; beta-catenin binding; ATP binding; armadillo repeat domain binding | {Autism, susceptibility to, 18} (615032 ) | (De Rubeis et al., 2014; Iossifov et al., 2014; Lim et al., 2017; Merico et al., 2017; O’Roak et al., 2012b) |
| ***DYRK1A*** | s1 | 21q22.13 | transferase activity; tau protein binding; protein tyrosine kinase activity; protein serine/threonine/tyrosine kinase activity; protein serine/threonine kinase activity; protein self-association; protein kinase activity; protein binding; nucleotide binding; non-membrane spanning protein tyrosine kinase activity; kinase activity; identical protein binding; ATP binding | Mental retardation, autosomal dominant 7 (614104) | (De Rubeis et al., 2014; Iossifov et al., 2012, 2014; Merico et al., 2017; O’Roak et al., 2012b) |
| ***KMT2A*** | s1 | 11q23.3 | zinc ion binding; unmethylated CpG binding; transferase activity; transcription regulatory region DNA binding; transcription factor activity, sequence-specific DNA binding; protein homodimerization activity; protein binding; methyltransferase activity; metal ion binding; lysine-acetylated histone binding; identical protein binding; histone-lysine N-methyltransferase activity; histone methyltransferase activity (H3-K4 specific); DNA binding; core promoter sequence-specific DNA binding; chromatin binding; AT DNA binding | Leukemia, myeloid/lymphoid or mixed-lineage (159555), Wiedemann-Steiner syndrome (605130) | (De Rubeis et al., 2014; Iossifov et al., 2014; Merico et al., 2017) |
| ***POGZ*** | s1 | 1q21.3 | protein binding; nucleic acid binding; metal ion binding; DNA binding | White-Sutton syndrome (616364) | (De Rubeis et al., 2014; Iossifov et al., 2012; Krumm et al., 2015; Merico et al., 2017; Neale et al., 2012) |
| ***PTEN*** | s1 | 10q23.31 | ubiquitin-specific protease binding; protein tyrosine/serine/threonine phosphatase activity; protein tyrosine phosphatase activity; protein tyrosine kinase binding; protein serine/threonine phosphatase activity; protein kinase binding; protein binding; platelet-derived growth factor receptor binding; phosphoprotein phosphatase activity; phosphatidylinositol-3-phosphatase activity; phosphatidylinositol-3,4-bisphosphate 3-phosphatase activity; phosphatidylinositol-3,4,5-trisphosphate 3-phosphatase activity; PDZ domain binding; magnesium ion binding; lipid binding; ionotropic glutamate receptor binding; inositol-1,3,4,5-tetrakisphosphate 3-phosphatase activity; identical protein binding; hydrolase activity; enzyme binding; anaphase-promoting complex binding | Bannayan-Riley-Ruvalcaba syndrome ( 153480), Cowden syndrome 1 (158350), Endometrial carcinoma, somatic (608089), Lhermitte-Duclos syndrome (158350), Macrocephaly/autism syndrome (605309), Malignant melanoma, somatic (155600), PTEN hamartoma tumor syndrome, Squamous cell carcinoma, head and neck, somatic (275355), VATER association with macrocephaly and ventriculomegaly (276950), {Glioma susceptibility 2} (613028), {Meningioma} (607174), {Prostate cancer, somatic} (176807) | (De Rubeis et al., 2014; Krumm et al., 2015; Merico et al., 2017; O’Roak et al., 2012b) |
| ***SETD5*** | s1 | 3p25.3 |  | Mental retardation, autosomal dominant 23 (615761) | (De Rubeis et al., 2014; Iossifov et al., 2012; Neale et al., 2012; Pinto et al., 2014) |
| ***SHANK3*** | s1 | 22q13.33 | scaffold protein binding; protein C-terminus binding; ionotropic glutamate receptor binding | Phelan-McDermid syndrome (606232), {Schizophrenia 15} (613950) | (De Rubeis et al., 2014; Lim et al., 2017; Merico et al., 2017) |
| ***SYNGAP1*** | s1 | 6p21.32 | SH3 domain binding; GTPase activator activity | Mental retardation, autosomal dominant 5 (612621) | (De Rubeis et al., 2014; Willsey et al., 2013) |
| ***TRIP12*** | s1 | 2q36.3 | ubiquitin-protein transferase activity; ubiquitin protein ligase activity; transferase activity; thyroid hormone receptor binding; protein binding | Mental retardation, autosomal dominant 49 (617752) | (Iossifov et al., 2012; Lim et al., 2017; Pinto et al., 2014) |
| ***ANKRD11*** | s 2 | 16q24.3 |  | KBG syndrome (148050) | (Iossifov et al., 2014; Merico et al., 2017) |
| ***BCL11A*** | s 2 | 2p16.1 | transcriptional repressor activity, RNA polymerase II core promoter proximal region sequence-specific binding; RNA polymerase II core promoter proximal region sequence-specific DNA binding; protein kinase binding; nucleic acid binding; metal ion binding | Dias-Logan syndrome (617101) | (De Rubeis et al., 2014; Iossifov et al., 2012) |
| ***CHD2*** | s 2 | 15q26.1 | RNA binding; protein binding; nucleotide binding; hydrolase activity; histone binding; helicase activity; DNA binding; core promoter sequence-specific DNA binding; ATP-dependent DNA helicase activity; ATP binding | Epileptic encephalopathy, childhood-onset (615369) | (De Rubeis et al., 2014; Iossifov et al., 2014; Lim et al., 2017; Merico et al., 2017; Neale et al., 2012; Pinto et al., 2014) |
| ***CIC*** | s 2 | 19q13.2 | protein binding; DNA binding; chromatin binding | Mental retardation, autosomal dominant 45 (617600) | (Iossifov et al., 2014; Merico et al., 2017) |
| ***CNTNAP2*** | s 2 | 7q35-q36.1 | protein binding; enzyme binding | Cortical dysplasia-focal epilepsy syndrome (610042), Pitt-Hopkins like syndrome 1 (610042), {Autism susceptibility 15} (612100) | (Anney et al., 2010; O’Roak et al., 2012a) |
| ***DDX3X*** | s 2 | Xp11.4 | translation initiation factor binding; transcription factor binding; RNA strand annealing activity; RNA stem-loop binding; RNA binding; ribosomal small subunit binding; protein serine/threonine kinase activator activity; protein binding; poly(A) binding; nucleotide binding; nucleoside-triphosphatase activity; nucleic acid binding; mRNA 5'-UTR binding; hydrolase activity; helicase activity; GTPase activity; eukaryotic initiation factor 4E binding; DNA binding; CTPase activity; cadherin binding; ATPase activity; ATP-dependent RNA helicase activity; ATP-dependent DNA helicase activity; ATP binding | Mental retardation, X-linked 102 (300958) | (Iossifov et al., 2014; Merico et al., 2017) |
| ***DEAF1*** | s 2 | 11p15.5 | transcriptional repressor activity, RNA polymerase II transcription regulatory region sequence-specific binding I; transcription factor activity, sequence-specific DNA binding; RNA polymerase II regulatory region sequence-specific DNA binding; protein binding; metal ion binding; DNA binding | ?Dyskinesia, seizures, and intellectual developmental disorder (617171), Mental retardation, autosomal dominant 24 (615828) | (De Rubeis et al., 2014) |
| ***FOXP1*** | s 2 | 3p13 | transcription regulatory region DNA binding; transcription factor binding; transcription factor activity, sequence-specific DNA binding; transcription factor activity, RNA polymerase II distal enhancer sequence-specific binding; sequence-specific DNA binding; RNA polymerase II transcription factor activity, sequence-specific DNA binding; RNA polymerase II core promoter proximal region sequence-specific DNA binding; protein self-association; protein homodimerization activity; protein heterodimerization activity; protein binding; metal ion binding; DNA binding; chromatin binding; androgen receptor binding | Mental retardation with language impairment and with or without autistic features (613670) | (Iossifov et al., 2014; Lim et al., 2017; Merico et al., 2017; O’Roak et al., 2012b) |
| ***MAGEL2*** | s 2 | 15q11.2 | ubiquitin-protein transferase activity; protein binding | Schaaf-Yang syndrome (615547) | (Schaaf et al., 2013) |
| ***MBOAT7*** | s 2 | 19q13.42 | transferase activity, transferring acyl groups; transferase activity; protein binding; lysophospholipid acyltransferase activity; 2-acylglycerol-3-phosphate O-acyltransferase activity; 1-acylglycerol-3-phosphate O-acyltransferase activity | Mental retardation, autosomal recessive 57 (617188) | (De Rubeis et al., 2014) |
| ***MECP2*** | s 2 | Xq28 | transcriptional repressor activity, RNA polymerase II core promoter proximal region sequence-specific binding; transcription factor binding; transcription factor activity, sequence-specific DNA binding; transcription corepressor activity; siRNA binding; RNA binding; protein N-terminus binding; protein domain specific binding; protein binding; mRNA binding; methyl-CpG binding; double-stranded methylated DNA binding | Encephalopathy, neonatal severe (300673), Mental retardation, X-linked syndromic, Lubs type (300260), Mental retardation, X-linked, syndromic 13 (300055), Rett syndrome (312750), Rett syndrome, atypical (312750), Rett syndrome, preserved speech variant (312750), {Autism susceptibility, X-linked 3} (300496) | (Merico et al., 2017) |
| ***UPF3B*** | s 2 | Xq24 | RNA binding; protein binding; nucleocytoplasmic transporter activity; nucleic acid binding; mRNA binding | Mental retardation, X-linked, syndromic 14 (300676) | (Iossifov et al., 2014; Merico et al., 2017) |
| ***WAC*** | s 2 | 10p12.1 | RNA polymerase II core binding; protein binding; chromatin binding | Desanto-Shinawi syndrome (616708) | (Iossifov et al., 2014; Merico et al., 2017) |

**Supplementary Table 2.** Gene-gene interactions reported by GeneMANIA with those genes carrying DNMs included in SFARI with scores 1 and 2 employed to create the network represented in Figure 1. Weight, source of network information and the selected network employed to create the interaction are represented.

| Gene 1 | Gene 2 | Weight | Network group | Network |
| --- | --- | --- | --- | --- |
| KMT2C | UBN2 | 0.008907883 | Co-expression | Bahr-Bowler-2013 |
| INTS6 | KMT2C | 0.009578812 | Co-expression | Bahr-Bowler-2013 |
| ASH1L | UBN2 | 0.009974718 | Co-expression | Bahr-Bowler-2013 |
| NAA15 | INTS6 | 0.012641064 | Co-expression | Bahr-Bowler-2013 |
| MED13L | UBN2 | 0.011041215 | Co-expression | Bahr-Bowler-2013 |
| MED13L | KMT2C | 0.01975944 | Co-expression | Bahr-Bowler-2013 |
| TNRC6B | UBN2 | 0.006566489 | Co-expression | Bahr-Bowler-2013 |
| TNRC6B | KMT2C | 0.010946862 | Co-expression | Bahr-Bowler-2013 |
| TNRC6B | ASH1L | 0.013503585 | Co-expression | Bahr-Bowler-2013 |
| KDM5B | GIGYF2 | 0.013121913 | Co-expression | Bahr-Bowler-2013 |
| WDFY3 | MED13L | 0.005751591 | Co-expression | Bahr-Bowler-2013 |
| MED13 | UBN2 | 0.010078115 | Co-expression | Bahr-Bowler-2013 |
| MED13 | KMT2C | 0.014431559 | Co-expression | Bahr-Bowler-2013 |
| MED13 | TBL1XR1 | 0.022210617 | Co-expression | Bahr-Bowler-2013 |
| ILF2 | IRF2BPL | 0.019355837 | Co-expression | Bahr-Bowler-2013 |
| CUL3 | TBL1XR1 | 0.016213536 | Co-expression | Bahr-Bowler-2013 |
| UBN1 | KMT2C | 0.008782483 | Co-expression | Bahr-Bowler-2013 |
| MED12 | SMARCC2 | 0.0158279 | Co-expression | Bahr-Bowler-2013 |
| MYT1L | SHANK2 | 0.013354452 | Co-expression | Wang-Maris-2006 |
| SCN9A | ANK2 | 0.0091171255 | Co-expression | Wang-Maris-2006 |
| GRIA1 | MYT1L | 0.012266724 | Co-expression | Wang-Maris-2006 |
| NRXN1 | ANK2 | 0.01563374 | Co-expression | Wang-Maris-2006 |
| NRXN1 | GRIA1 | 0.015987033 | Co-expression | Wang-Maris-2006 |
| TNRC6B | TCF20 | 0.013869711 | Co-expression | Wang-Maris-2006 |
| CTNND2 | GRIA1 | 0.0218302 | Co-expression | Wang-Maris-2006 |
| SCN2A | SCN9A | 0.004255575 | Co-expression | Wang-Maris-2006 |
| SCN2A | GRIA1 | 0.009872705 | Co-expression | Wang-Maris-2006 |
| TBR1 | SLC6A1 | 0.008557321 | Co-expression | Wang-Maris-2006 |
| KDM5B | TCF20 | 0.017740238 | Co-expression | Wang-Maris-2006 |
| USP7 | GIGYF2 | 0.011568467 | Co-expression | Wang-Maris-2006 |
| PHF3 | MED13L | 0.012056054 | Co-expression | Wang-Maris-2006 |
| PHF3 | WDFY3 | 0.007389789 | Co-expression | Wang-Maris-2006 |
| SRSF11 | MED13L | 0.012979265 | Co-expression | Wang-Maris-2006 |
| SRSF11 | PHF3 | 0.0068400726 | Co-expression | Wang-Maris-2006 |
| CUL3 | GIGYF2 | 0.01240512 | Co-expression | Wang-Maris-2006 |
| CUL3 | WDFY3 | 0.009314236 | Co-expression | Wang-Maris-2006 |
| MYT1 | GABRB3 | 0.010188389 | Co-expression | Wang-Maris-2006 |
| MYT1 | SCN2A | 0.005953512 | Co-expression | Wang-Maris-2006 |
| GRIA2 | MYT1L | 0.010083766 | Co-expression | Wang-Maris-2006 |
| GRIA2 | TNRC6B | 0.011389478 | Co-expression | Wang-Maris-2006 |
| GRIA2 | SCN2A | 0.0066694138 | Co-expression | Wang-Maris-2006 |
| GRIA2 | WDFY3 | 0.012431782 | Co-expression | Wang-Maris-2006 |
| DST | SCN9A | 0.009187257 | Co-expression | Wang-Maris-2006 |
| DST | SCN2A | 0.008241924 | Co-expression | Wang-Maris-2006 |
| ASH1L | DIP2C | 0.01786314 | Co-expression | Mallon-McKay-2013 |
| CACNA1H | IRF2BPL | 0.01884355 | Co-expression | Mallon-McKay-2013 |
| SLC6A1 | MYT1L | 0.009409471 | Co-expression | Mallon-McKay-2013 |
| GRIA1 | MYT1L | 0.0076554245 | Co-expression | Mallon-McKay-2013 |
| SPAST | INTS6 | 0.03273186 | Co-expression | Mallon-McKay-2013 |
| NRXN1 | CACNA2D3 | 0.012142438 | Co-expression | Mallon-McKay-2013 |
| NRXN1 | MYT1L | 0.0048816586 | Co-expression | Mallon-McKay-2013 |
| SCN2A | MYT1L | 0.004014961 | Co-expression | Mallon-McKay-2013 |
| SCN2A | GRIA1 | 0.009363521 | Co-expression | Mallon-McKay-2013 |
| SCN2A | NRXN1 | 0.0060287016 | Co-expression | Mallon-McKay-2013 |
| GRIN2B | MYT1L | 0.0049865693 | Co-expression | Mallon-McKay-2013 |
| GRIN2B | GRIA1 | 0.012621141 | Co-expression | Mallon-McKay-2013 |
| GRIN2B | SCN2A | 0.0062553347 | Co-expression | Mallon-McKay-2013 |
| TBR1 | MYT1L | 0.005317564 | Co-expression | Mallon-McKay-2013 |
| TBR1 | SCN2A | 0.007053238 | Co-expression | Mallon-McKay-2013 |
| TBR1 | GRIN2B | 0.008972127 | Co-expression | Mallon-McKay-2013 |
| KDM5B | RANBP17 | 0.0091457125 | Co-expression | Mallon-McKay-2013 |
| MED13 | KMT2C | 0.02269232 | Co-expression | Mallon-McKay-2013 |
| PHF3 | TBL1XR1 | 0.008555559 | Co-expression | Mallon-McKay-2013 |
| PHF3 | TNRC6B | 0.008460463 | Co-expression | Mallon-McKay-2013 |
| SRSF11 | TCF20 | 0.0053750887 | Co-expression | Mallon-McKay-2013 |
| ILF2 | SPAST | 0.0067756763 | Co-expression | Mallon-McKay-2013 |
| CUL3 | PHF3 | 0.0071425554 | Co-expression | Mallon-McKay-2013 |
| MED12 | KMT2C | 0.010545376 | Co-expression | Mallon-McKay-2013 |
| MED12 | MED13L | 0.00973792 | Co-expression | Mallon-McKay-2013 |
| ST18 | MYT1L | 0.007534013 | Co-expression | Mallon-McKay-2013 |
| ST18 | SCN2A | 0.010471655 | Co-expression | Mallon-McKay-2013 |
| ST18 | TBR1 | 0.017712459 | Co-expression | Mallon-McKay-2013 |
| GRIA4 | MYT1 | 0.01751635 | Co-expression | Mallon-McKay-2013 |
| GRIA2 | MYT1L | 0.009009964 | Co-expression | Mallon-McKay-2013 |
| GRIA2 | SCN2A | 0.011366078 | Co-expression | Mallon-McKay-2013 |
| NRXN2 | NRXN1 | 0.007059619 | Co-expression | Mallon-McKay-2013 |
| NRXN2 | SCN4B | 0.010730434 | Co-expression | Mallon-McKay-2013 |
| CACNA1H | ANK2 | 0.033489857 | Co-expression | Burington-Shaughnessy-2008 |
| CTNND2 | SCN9A | 0.010080215 | Co-expression | Burington-Shaughnessy-2008 |
| SCN2A | DSCAM | 0.015952764 | Co-expression | Burington-Shaughnessy-2008 |
| TBR1 | GRIN2B | 0.014881447 | Co-expression | Burington-Shaughnessy-2008 |
| WDFY3 | DIP2C | 0.01807726 | Co-expression | Burington-Shaughnessy-2008 |
| WDFY3 | SCN9A | 0.017587915 | Co-expression | Burington-Shaughnessy-2008 |
| PHF3 | MED13L | 0.019933298 | Co-expression | Burington-Shaughnessy-2008 |
| CUL3 | NRXN1 | 0.018338915 | Co-expression | Burington-Shaughnessy-2008 |
| NCKAP1L | SMARCC2 | 0.021165846 | Co-expression | Burington-Shaughnessy-2008 |
| CD2BP2 | UBN1 | 0.024801197 | Co-expression | Burington-Shaughnessy-2008 |
| ST18 | CTNND2 | 0.007843815 | Co-expression | Burington-Shaughnessy-2008 |
| ST18 | WDFY3 | 0.008976232 | Co-expression | Burington-Shaughnessy-2008 |
| CACNA1H | CACNA1D | 0.01397464 | Co-expression | Wu-Garvey-2007 |
| SPAST | MED13L | 0.006861659 | Co-expression | Wu-Garvey-2007 |
| DSCAM | GRIA1 | 0.0130600035 | Co-expression | Wu-Garvey-2007 |
| TNRC6B | GIGYF2 | 0.022166356 | Co-expression | Wu-Garvey-2007 |
| GRIN2B | SLC6A1 | 0.012355963 | Co-expression | Wu-Garvey-2007 |
| TBR1 | CTNND2 | 0.006892522 | Co-expression | Wu-Garvey-2007 |
| PHF3 | MED13L | 0.005674113 | Co-expression | Wu-Garvey-2007 |
| KAT2B | MET | 0.016742969 | Co-expression | Wu-Garvey-2007 |
| SRSF11 | MED13L | 0.002846203 | Co-expression | Wu-Garvey-2007 |
| CUL3 | KAT2B | 0.0102848485 | Co-expression | Wu-Garvey-2007 |
| MED12 | BCKDK | 0.013393021 | Co-expression | Wu-Garvey-2007 |
| ST18 | MYT1L | 0.008537529 | Co-expression | Wu-Garvey-2007 |
| NRXN2 | NRXN3 | 0.017559506 | Co-expression | Wu-Garvey-2007 |
| ASH1L | DIP2C | 0.025702816 | Co-expression | Chen-Brown-2002 |
| SLC6A1 | SHANK2 | 0.012703756 | Co-expression | Chen-Brown-2002 |
| DSCAM | NRXN1 | 0.0075084963 | Co-expression | Chen-Brown-2002 |
| KDM6A | MED13L | 0.014618278 | Co-expression | Chen-Brown-2002 |
| PHF3 | MED13L | 0.013260207 | Co-expression | Chen-Brown-2002 |
| SRSF11 | MED13L | 0.0121158995 | Co-expression | Chen-Brown-2002 |
| SRSF11 | KDM6A | 0.0155297965 | Co-expression | Chen-Brown-2002 |
| UBN1 | NLGN3 | 0.02168759 | Co-expression | Chen-Brown-2002 |
| NCKAP1L | ANK2 | 0.0108584855 | Co-expression | Chen-Brown-2002 |
| DST | PHF3 | 0.017744346 | Co-expression | Chen-Brown-2002 |
| ANK2 | DIP2C | 0.027714863 | Co-expression | Roth-Zlotnik-2006 |
| DSCAM | SLC6A1 | 0.007089582 | Co-expression | Roth-Zlotnik-2006 |
| KDM6A | KMT2C | 0.028013967 | Co-expression | Roth-Zlotnik-2006 |
| RIMS1 | PTCHD1 | 0.011738146 | Co-expression | Roth-Zlotnik-2006 |
| CTNND2 | SLC6A1 | 0.01231086 | Co-expression | Roth-Zlotnik-2006 |
| SCN2A | PTCHD1 | 0.0075539183 | Co-expression | Roth-Zlotnik-2006 |
| SCN2A | GRIA1 | 0.006199577 | Co-expression | Roth-Zlotnik-2006 |
| SCN2A | NRXN1 | 0.010399918 | Co-expression | Roth-Zlotnik-2006 |
| SCN2A | RIMS1 | 0.0041704574 | Co-expression | Roth-Zlotnik-2006 |
| RELN | RIMS1 | 0.008509801 | Co-expression | Roth-Zlotnik-2006 |
| SCN4B | CACNA2D3 | 0.0073228073 | Co-expression | Roth-Zlotnik-2006 |
| NRXN3 | PTCHD1 | 0.011919006 | Co-expression | Roth-Zlotnik-2006 |
| NRXN3 | RIMS1 | 0.005729331 | Co-expression | Roth-Zlotnik-2006 |
| NRXN3 | SCN2A | 0.0040660677 | Co-expression | Roth-Zlotnik-2006 |
| NRXN3 | RELN | 0.007716751 | Co-expression | Roth-Zlotnik-2006 |
| NRXN2 | SCN2A | 0.0042467844 | Co-expression | Roth-Zlotnik-2006 |
| NRXN1 | GABRB3 | 0.006934312 | Co-expression | Bild-Nevins-2006 B |
| DSCAM | SLC6A1 | 0.011191085 | Co-expression | Bild-Nevins-2006 B |
| TNRC6B | TCF20 | 0.018049965 | Co-expression | Bild-Nevins-2006 B |
| CTNND2 | NRXN1 | 0.005172923 | Co-expression | Bild-Nevins-2006 B |
| SCN2A | SCN9A | 0.005442085 | Co-expression | Bild-Nevins-2006 B |
| SCN2A | GRIA1 | 0.0060237055 | Co-expression | Bild-Nevins-2006 B |
| SCN2A | DSCAM | 0.004098024 | Co-expression | Bild-Nevins-2006 B |
| SCN2A | RIMS1 | 0.005855085 | Co-expression | Bild-Nevins-2006 B |
| WDFY3 | GIGYF2 | 0.010332577 | Co-expression | Bild-Nevins-2006 B |
| RELN | SCN9A | 0.011804704 | Co-expression | Bild-Nevins-2006 B |
| RELN | SCN2A | 0.004002466 | Co-expression | Bild-Nevins-2006 B |
| SRSF11 | SPAST | 0.009149326 | Co-expression | Bild-Nevins-2006 B |
| ST18 | NRXN1 | 0.006534621 | Co-expression | Bild-Nevins-2006 B |
| NRXN2 | RELN | 0.010632013 | Co-expression | Bild-Nevins-2006 B |
| ASH1L | TCF20 | 0.0057034534 | Co-expression | Dobbin-Giordano-2005 |
| MED13L | KMT5B | 0.017430635 | Co-expression | Dobbin-Giordano-2005 |
| RIMS1 | NLGN3 | 0.003170006 | Co-expression | Dobbin-Giordano-2005 |
| RIMS1 | GRIP1 | 0.001519433 | Co-expression | Dobbin-Giordano-2005 |
| SCN2A | CACNA1D | 0.02305361 | Co-expression | Dobbin-Giordano-2005 |
| TBR1 | ASH1L | 0.005454799 | Co-expression | Dobbin-Giordano-2005 |
| MED13 | KMT5B | 0.016418274 | Co-expression | Dobbin-Giordano-2005 |
| ILF2 | TBL1XR1 | 0.0121416785 | Co-expression | Dobbin-Giordano-2005 |
| CUL3 | SPAST | 0.028929235 | Co-expression | Dobbin-Giordano-2005 |
| MYT1 | GABRB3 | 0.017837053 | Co-expression | Dobbin-Giordano-2005 |
| MED28 | TBL1XR1 | 0.014974633 | Co-expression | Dobbin-Giordano-2005 |
| MED28 | ERBIN | 0.009182728 | Co-expression | Dobbin-Giordano-2005 |
| MED28 | ILF2 | 0.009165671 | Co-expression | Dobbin-Giordano-2005 |
| GRIA2 | ANK2 | 0.010282978 | Co-expression | Dobbin-Giordano-2005 |
| DIP2C | RANBP17 | 0.008873917 | Co-expression | Perou-Botstein-2000 |
| CACNA1D | SRCAP | 0.0113258455 | Co-expression | Perou-Botstein-2000 |
| GRIA1 | MED13L | 0.017051458 | Co-expression | Perou-Botstein-2000 |
| KAT2B | PHF3 | 0.0157982 | Co-expression | Perou-Botstein-2000 |
| SRSF11 | KMT2C | 0.0115938755 | Co-expression | Perou-Botstein-2000 |
| GRIA4 | RANBP17 | 0.00769782 | Co-expression | Perou-Botstein-2000 |
| GRIA2 | MED13L | 0.018174756 | Co-expression | Perou-Botstein-2000 |
| GIGYF2 | IRF2BPL | 0.013931686 | Co-expression | Innocenti-Brown-2011 |
| CACNA1H | CACNA1D | 0.022447899 | Co-expression | Innocenti-Brown-2011 |
| MED13L | TCF20 | 0.010441152 | Co-expression | Innocenti-Brown-2011 |
| CTNND2 | GABRB3 | 0.015691008 | Co-expression | Innocenti-Brown-2011 |
| USP7 | SRCAP | 0.009295484 | Co-expression | Innocenti-Brown-2011 |
| SRSF11 | SRCAP | 0.0053977203 | Co-expression | Innocenti-Brown-2011 |
| SRSF11 | ERBIN | 0.010201851 | Co-expression | Innocenti-Brown-2011 |
| ILF2 | SRSF11 | 0.0073226774 | Co-expression | Innocenti-Brown-2011 |
| IRF2BP2 | PHF3 | 0.014687874 | Co-expression | Innocenti-Brown-2011 |
| ST18 | GRIP1 | 0.011117785 | Co-expression | Innocenti-Brown-2011 |
| SCN4B | CACNA2D3 | 0.008403427 | Co-expression | Innocenti-Brown-2011 |
| SCN4B | CTNND2 | 0.007750113 | Co-expression | Innocenti-Brown-2011 |
| NRXN3 | SCN4B | 0.008363876 | Co-expression | Innocenti-Brown-2011 |
| GRIA4 | ANK2 | 0.013631503 | Co-expression | Innocenti-Brown-2011 |
| IRF2BP1 | SRCAP | 0.0055573382 | Co-expression | Innocenti-Brown-2011 |
| NCKAP1 | TBL1XR1 | 0.015387185 | Co-expression | Wang-Cheung-2015 |
| TNRC6B | ERBIN | 0.014617795 | Co-expression | Wang-Cheung-2015 |
| GRIN2B | RIMS1 | 0.016605077 | Co-expression | Wang-Cheung-2015 |
| KAT2B | KMT5B | 0.0052504707 | Co-expression | Wang-Cheung-2015 |
| KAT2B | ERBIN | 0.005421652 | Co-expression | Wang-Cheung-2015 |
| KAT2B | WDFY3 | 0.0105083585 | Co-expression | Wang-Cheung-2015 |
| KAT2B | SMARCC2 | 0.004853677 | Co-expression | Wang-Cheung-2015 |
| INTS6 | KMT5B | 0.0036025255 | Co-expression | Smirnov-Cheung-2009 |
| SLC6A1 | GRIP1 | 0.024677532 | Co-expression | Smirnov-Cheung-2009 |
| DSCAM | CACNA1H | 0.021231793 | Co-expression | Smirnov-Cheung-2009 |
| MED13 | KMT5B | 0.0057157595 | Co-expression | Smirnov-Cheung-2009 |
| PHF3 | ERBIN | 0.015382717 | Co-expression | Smirnov-Cheung-2009 |
| ILF2 | TBL1XR1 | 0.0024110975 | Co-expression | Smirnov-Cheung-2009 |
| USP7 | TNRC6B | 0.018477151 | Co-expression | Alizadeh-Staudt-2000 |
| KDM5B | MED13L | 0.014875708 | Co-expression | Rieger-Chu-2004 |
| RELN | CACNA1D | 0.011985586 | Co-expression | Rieger-Chu-2004 |
| USP7 | GIGYF2 | 0.013989381 | Co-expression | Rieger-Chu-2004 |
| SRSF11 | PHF3 | 0.009887132 | Co-expression | Rieger-Chu-2004 |
| NCKAP1L | TNRC6B | 0.0062272414 | Co-expression | Rieger-Chu-2004 |
| NCKAP1L | PHF3 | 0.0081671495 | Co-expression | Rieger-Chu-2004 |
| IRF2BP1 | GABRB3 | 0.0067331414 | Co-expression | Rieger-Chu-2004 |
| GRIA2 | SLC6A1 | 0.025529208 | Co-expression | Rieger-Chu-2004 |
| GRIA2 | DSCAM | 0.019566441 | Co-expression | Rieger-Chu-2004 |
| GRIA2 | CTNND2 | 0.020588705 | Co-expression | Rieger-Chu-2004 |
| ANK2 | NLGN3 | 0.016306605 | Co-localization | Johnson-Shoemaker-2003 |
| ASH1L | ANK2 | 0.015427804 | Co-localization | Johnson-Shoemaker-2003 |
| NCKAP1 | ASH1L | 0.019102447 | Co-localization | Johnson-Shoemaker-2003 |
| NRXN1 | ANK2 | 0.012943148 | Co-localization | Johnson-Shoemaker-2003 |
| SCN2A | NRXN1 | 0.016705144 | Co-localization | Johnson-Shoemaker-2003 |
| GRIN2B | GRIA1 | 0.016678855 | Co-localization | Johnson-Shoemaker-2003 |
| MED13 | KDM6A | 0.021070872 | Co-localization | Johnson-Shoemaker-2003 |
| RELN | SCN2A | 0.0105959205 | Co-localization | Johnson-Shoemaker-2003 |
| GRIA2 | CACNA2D3 | 0.012501432 | Co-localization | Johnson-Shoemaker-2003 |
| GRIA2 | NLGN3 | 0.01563167 | Co-localization | Johnson-Shoemaker-2003 |
| GRIA2 | GRIA1 | 0.009917008 | Co-localization | Johnson-Shoemaker-2003 |
| GRIA2 | SCN2A | 0.010785489 | Co-localization | Johnson-Shoemaker-2003 |
| GRIA2 | NRXN3 | 0.017990466 | Co-localization | Johnson-Shoemaker-2003 |
| NRXN2 | ANK2 | 0.017004702 | Co-localization | Johnson-Shoemaker-2003 |
| NRXN2 | NRXN1 | 0.024914535 | Co-localization | Johnson-Shoemaker-2003 |
| NRXN2 | SCN2A | 0.020540738 | Co-localization | Johnson-Shoemaker-2003 |
| NRXN2 | GRIA4 | 0.020456415 | Co-localization | Johnson-Shoemaker-2003 |
| DST | ANK2 | 0.013796837 | Co-localization | Johnson-Shoemaker-2003 |
| DST | GRIA1 | 0.014812263 | Co-localization | Johnson-Shoemaker-2003 |
| DST | NCKAP1 | 0.016457543 | Co-localization | Johnson-Shoemaker-2003 |
| CTNND2 | GABRB3 | 0.006225095 | Co-localization | Schadt-Shoemaker-2004 |
| CTNND2 | DSCAM | 0.010918354 | Co-localization | Schadt-Shoemaker-2004 |
| GRIN2B | GABRB3 | 0.008328055 | Co-localization | Schadt-Shoemaker-2004 |
| GRIN2B | DSCAM | 0.01114187 | Co-localization | Schadt-Shoemaker-2004 |
| GRIN2B | CTNND2 | 0.006659904 | Co-localization | Schadt-Shoemaker-2004 |
| GRIA4 | DSCAM | 0.01167741 | Co-localization | Schadt-Shoemaker-2004 |
| GRIA4 | CTNND2 | 0.0062861103 | Co-localization | Schadt-Shoemaker-2004 |
| GRIA4 | GRIN2B | 0.006422703 | Co-localization | Schadt-Shoemaker-2004 |
| GRIA2 | GABRB3 | 0.0075816154 | Co-localization | Schadt-Shoemaker-2004 |
| GRIA2 | DSCAM | 0.01119408 | Co-localization | Schadt-Shoemaker-2004 |
| GRIA2 | CTNND2 | 0.005978434 | Co-localization | Schadt-Shoemaker-2004 |
| GRIA2 | GRIN2B | 0.006316649 | Co-localization | Schadt-Shoemaker-2004 |
| GRIA2 | GRIA4 | 0.007023969 | Co-localization | Schadt-Shoemaker-2004 |
| DST | CTNND2 | 0.010191792 | Co-localization | Schadt-Shoemaker-2004 |
| CACNA2D3 | CNTN4 | 0.00019954208 | Genetic Interactions | Lin-Smith-2010 |
| IRF2BPL | CNTN4 | 0.00053538184 | Genetic Interactions | Lin-Smith-2010 |
| KMT5B | CACNA2D3 | 0.00041130272 | Genetic Interactions | Lin-Smith-2010 |
| KMT2C | CNTN4 | 0.0003396518 | Genetic Interactions | Lin-Smith-2010 |
| KMT2C | CACNA2D3 | 0.00038595265 | Genetic Interactions | Lin-Smith-2010 |
| KMT2C | KMT5B | 0.00070010155 | Genetic Interactions | Lin-Smith-2010 |
| TCF20 | CNTN4 | 0.00053560664 | Genetic Interactions | Lin-Smith-2010 |
| TCF20 | CACNA2D3 | 0.0006086198 | Genetic Interactions | Lin-Smith-2010 |
| INTS6 | RANBP17 | 0.0010033273 | Genetic Interactions | Lin-Smith-2010 |
| INTS6 | IRF2BPL | 0.0011523789 | Genetic Interactions | Lin-Smith-2010 |
| INTS6 | KMT2C | 0.0007310811 | Genetic Interactions | Lin-Smith-2010 |
| DIP2C | KATNAL2 | 0.0007907327 | Genetic Interactions | Lin-Smith-2010 |
| DIP2C | KMT2C | 0.00064802286 | Genetic Interactions | Lin-Smith-2010 |
| SHANK2 | CNTN4 | 0.00033755577 | Genetic Interactions | Lin-Smith-2010 |
| SHANK2 | CACNA2D3 | 0.00038357094 | Genetic Interactions | Lin-Smith-2010 |
| SHANK2 | DIP2C | 0.0006440239 | Genetic Interactions | Lin-Smith-2010 |
| ANK2 | SRCAP | 0.0018464098 | Genetic Interactions | Lin-Smith-2010 |
| ANK2 | KMT2C | 0.0004351461 | Genetic Interactions | Lin-Smith-2010 |
| ANK2 | INTS6 | 0.00048424726 | Genetic Interactions | Lin-Smith-2010 |
| ANK2 | SHANK2 | 0.00043246074 | Genetic Interactions | Lin-Smith-2010 |
| CACNA1D | KMT2C | 0.0007039691 | Genetic Interactions | Lin-Smith-2010 |
| CACNA1D | TCF20 | 0.001110109 | Genetic Interactions | Lin-Smith-2010 |
| CACNA1D | ANK2 | 0.00046628906 | Genetic Interactions | Lin-Smith-2010 |
| MYT1L | CNTN4 | 0.0003356048 | Genetic Interactions | Lin-Smith-2010 |
| MYT1L | SHANK2 | 0.00064511824 | Genetic Interactions | Lin-Smith-2010 |
| SCN9A | SHANK2 | 0.000999395 | Genetic Interactions | Lin-Smith-2010 |
| SCN9A | ANK2 | 0.00066608127 | Genetic Interactions | Lin-Smith-2010 |
| SCN9A | CACNA1D | 0.0010775706 | Genetic Interactions | Lin-Smith-2010 |
| ASH1L | PTCHD1 | 0.0009561279 | Genetic Interactions | Lin-Smith-2010 |
| ASH1L | CACNA2D3 | 0.00026673402 | Genetic Interactions | Lin-Smith-2010 |
| ASH1L | IRF2BPL | 0.0007156614 | Genetic Interactions | Lin-Smith-2010 |
| ASH1L | KMT5B | 0.00048384402 | Genetic Interactions | Lin-Smith-2010 |
| ASH1L | KMT2C | 0.000454023 | Genetic Interactions | Lin-Smith-2010 |
| ASH1L | CACNA1D | 0.00048651698 | Genetic Interactions | Lin-Smith-2010 |
| TBL1XR1 | CNTN4 | 0.00019546502 | Genetic Interactions | Lin-Smith-2010 |
| TBL1XR1 | ANK2 | 0.00025042068 | Genetic Interactions | Lin-Smith-2010 |
| TBL1XR1 | MYT1L | 0.00037356207 | Genetic Interactions | Lin-Smith-2010 |
| GIGYF2 | CNTN4 | 0.00045246969 | Genetic Interactions | Lin-Smith-2010 |
| GIGYF2 | DIP2C | 0.0008632685 | Genetic Interactions | Lin-Smith-2010 |
| GIGYF2 | ASH1L | 0.0006048301 | Genetic Interactions | Lin-Smith-2010 |
| NAA15 | CNTN4 | 0.00037613435 | Genetic Interactions | Lin-Smith-2010 |
| NAA15 | CACNA2D3 | 0.0004274085 | Genetic Interactions | Lin-Smith-2010 |
| NAA15 | KMT5B | 0.00077530066 | Genetic Interactions | Lin-Smith-2010 |
| NAA15 | DIP2C | 0.0007176281 | Genetic Interactions | Lin-Smith-2010 |
| NAA15 | SHANK2 | 0.00072302646 | Genetic Interactions | Lin-Smith-2010 |
| NAA15 | MYT1L | 0.00071884756 | Genetic Interactions | Lin-Smith-2010 |
| GABRB3 | KATNAL2 | 0.0005975283 | Genetic Interactions | Lin-Smith-2010 |
| GABRB3 | CNTN4 | 0.0002531743 | Genetic Interactions | Lin-Smith-2010 |
| GABRB3 | INTS6 | 0.0005449432 | Genetic Interactions | Lin-Smith-2010 |
| GABRB3 | TBL1XR1 | 0.0002818086 | Genetic Interactions | Lin-Smith-2010 |
| GABRB3 | NAA15 | 0.0005422858 | Genetic Interactions | Lin-Smith-2010 |
| CACNA1H | TCF20 | 0.0040629427 | Genetic Interactions | Lin-Smith-2010 |
| CACNA1H | ANK2 | 0.0017065943 | Genetic Interactions | Lin-Smith-2010 |
| MED13L | INTS6 | 0.0006545421 | Genetic Interactions | Lin-Smith-2010 |
| MED13L | ANK2 | 0.00038958938 | Genetic Interactions | Lin-Smith-2010 |
| MED13L | MYT1L | 0.0005811654 | Genetic Interactions | Lin-Smith-2010 |
| MED13L | TBL1XR1 | 0.00033848593 | Genetic Interactions | Lin-Smith-2010 |
| MED13L | GABRB3 | 0.00043842083 | Genetic Interactions | Lin-Smith-2010 |
| MET | KATNAL2 | 0.0009312796 | Genetic Interactions | Lin-Smith-2010 |
| MET | ANK2 | 0.0005055247 | Genetic Interactions | Lin-Smith-2010 |
| MET | ASH1L | 0.00052745466 | Genetic Interactions | Lin-Smith-2010 |
| ERBIN | CNTN4 | 0.0003220794 | Genetic Interactions | Lin-Smith-2010 |
| GRIA1 | CACNA2D3 | 0.0002276292 | Genetic Interactions | Lin-Smith-2010 |
| GRIA1 | KMT5B | 0.0004129096 | Genetic Interactions | Lin-Smith-2010 |
| GRIA1 | TCF20 | 0.0006109975 | Genetic Interactions | Lin-Smith-2010 |
| GRIA1 | CACNA1D | 0.00041519065 | Genetic Interactions | Lin-Smith-2010 |
| GRIA1 | SCN9A | 0.00059308857 | Genetic Interactions | Lin-Smith-2010 |
| GRIA1 | GABRB3 | 0.00028881058 | Genetic Interactions | Lin-Smith-2010 |
| NCKAP1 | IRF2BPL | 0.0011434334 | Genetic Interactions | Lin-Smith-2010 |
| NCKAP1 | SRCAP | 0.003078039 | Genetic Interactions | Lin-Smith-2010 |
| NCKAP1 | KMT2C | 0.00072540605 | Genetic Interactions | Lin-Smith-2010 |
| NCKAP1 | INTS6 | 0.00080725964 | Genetic Interactions | Lin-Smith-2010 |
| NCKAP1 | DIP2C | 0.0007155467 | Genetic Interactions | Lin-Smith-2010 |
| NCKAP1 | SHANK2 | 0.0007209294 | Genetic Interactions | Lin-Smith-2010 |
| NCKAP1 | NAA15 | 0.00080332306 | Genetic Interactions | Lin-Smith-2010 |
| NCKAP1 | GRIA1 | 0.00042783382 | Genetic Interactions | Lin-Smith-2010 |
| SPAST | ANK2 | 0.0012467519 | Genetic Interactions | Lin-Smith-2010 |
| DSCAM | CNTN4 | 0.0003164852 | Genetic Interactions | Lin-Smith-2010 |
| DSCAM | INTS6 | 0.0006812163 | Genetic Interactions | Lin-Smith-2010 |
| DSCAM | DIP2C | 0.0006038232 | Genetic Interactions | Lin-Smith-2010 |
| DSCAM | ANK2 | 0.0004054661 | Genetic Interactions | Lin-Smith-2010 |
| DSCAM | ASH1L | 0.00042305546 | Genetic Interactions | Lin-Smith-2010 |
| DSCAM | MED13L | 0.00054805604 | Genetic Interactions | Lin-Smith-2010 |
| TNRC6B | KATNAL2 | 0.0012278383 | Genetic Interactions | Lin-Smith-2010 |
| TNRC6B | CNTN4 | 0.0005202383 | Genetic Interactions | Lin-Smith-2010 |
| TNRC6B | RANBP17 | 0.0013809531 | Genetic Interactions | Lin-Smith-2010 |
| TNRC6B | GRIA1 | 0.0005934659 | Genetic Interactions | Lin-Smith-2010 |
| RIMS1 | KATNAL2 | 0.00066752796 | Genetic Interactions | Lin-Smith-2010 |
| RIMS1 | CNTN4 | 0.00028283335 | Genetic Interactions | Lin-Smith-2010 |
| RIMS1 | CACNA2D3 | 0.0003213888 | Genetic Interactions | Lin-Smith-2010 |
| RIMS1 | TCF20 | 0.000862665 | Genetic Interactions | Lin-Smith-2010 |
| RIMS1 | DIP2C | 0.0005396187 | Genetic Interactions | Lin-Smith-2010 |
| RIMS1 | CACNA1D | 0.00058620604 | Genetic Interactions | Lin-Smith-2010 |
| RIMS1 | MYT1L | 0.0005405357 | Genetic Interactions | Lin-Smith-2010 |
| RIMS1 | ASH1L | 0.000378072 | Genetic Interactions | Lin-Smith-2010 |
| RIMS1 | ERBIN | 0.0005187513 | Genetic Interactions | Lin-Smith-2010 |
| RIMS1 | GRIA1 | 0.00032264437 | Genetic Interactions | Lin-Smith-2010 |
| RIMS1 | NCKAP1 | 0.00060405687 | Genetic Interactions | Lin-Smith-2010 |
| CTNND2 | CNTN4 | 0.00019283422 | Genetic Interactions | Lin-Smith-2010 |
| CTNND2 | RANBP17 | 0.0005118713 | Genetic Interactions | Lin-Smith-2010 |
| CTNND2 | TCF20 | 0.00058816024 | Genetic Interactions | Lin-Smith-2010 |
| CTNND2 | NLGN3 | 0.0018675072 | Genetic Interactions | Lin-Smith-2010 |
| CTNND2 | SHANK2 | 0.0003706767 | Genetic Interactions | Lin-Smith-2010 |
| CTNND2 | SCN9A | 0.0005709207 | Genetic Interactions | Lin-Smith-2010 |
| CTNND2 | ASH1L | 0.00025776742 | Genetic Interactions | Lin-Smith-2010 |
| CTNND2 | TBL1XR1 | 0.000214644 | Genetic Interactions | Lin-Smith-2010 |
| CTNND2 | GRIA1 | 0.00021997718 | Genetic Interactions | Lin-Smith-2010 |
| CTNND2 | NCKAP1 | 0.00041184266 | Genetic Interactions | Lin-Smith-2010 |
| SCN2A | CNTN4 | 0.00032436528 | Genetic Interactions | Lin-Smith-2010 |
| SCN2A | KMT5B | 0.0006685924 | Genetic Interactions | Lin-Smith-2010 |
| SCN2A | TBL1XR1 | 0.00036105135 | Genetic Interactions | Lin-Smith-2010 |
| SCN2A | GRIA1 | 0.00037002226 | Genetic Interactions | Lin-Smith-2010 |
| SCN2A | DSCAM | 0.00058459264 | Genetic Interactions | Lin-Smith-2010 |
| SCN2A | KDM6A | 0.0028250534 | Genetic Interactions | Lin-Smith-2010 |
| SCN2A | CTNND2 | 0.00035619191 | Genetic Interactions | Lin-Smith-2010 |
| GRIN2B | CACNA2D3 | 0.00028268778 | Genetic Interactions | Lin-Smith-2010 |
| GRIN2B | RANBP17 | 0.0006603643 | Genetic Interactions | Lin-Smith-2010 |
| GRIN2B | DIP2C | 0.00047463892 | Genetic Interactions | Lin-Smith-2010 |
| GRIN2B | MYT1L | 0.00047544547 | Genetic Interactions | Lin-Smith-2010 |
| GRIN2B | TBL1XR1 | 0.0002769119 | Genetic Interactions | Lin-Smith-2010 |
| GRIN2B | GABRB3 | 0.0003586676 | Genetic Interactions | Lin-Smith-2010 |
| GRIN2B | MED13L | 0.00043080281 | Genetic Interactions | Lin-Smith-2010 |
| GRIN2B | TNRC6B | 0.0007370125 | Genetic Interactions | Lin-Smith-2010 |
| KDM5B | CACNA2D3 | 0.0004341437 | Genetic Interactions | Lin-Smith-2010 |
| KDM5B | CACNA1D | 0.00079186854 | Genetic Interactions | Lin-Smith-2010 |
| KDM5B | ASH1L | 0.0005107135 | Genetic Interactions | Lin-Smith-2010 |
| KDM5B | MED13L | 0.00066161447 | Genetic Interactions | Lin-Smith-2010 |
| KDM5B | GRIA1 | 0.00043583984 | Genetic Interactions | Lin-Smith-2010 |
| WDFY3 | CNTN4 | 0.00029361644 | Genetic Interactions | Lin-Smith-2010 |
| WDFY3 | KMT2C | 0.00056791055 | Genetic Interactions | Lin-Smith-2010 |
| WDFY3 | TBL1XR1 | 0.0003268248 | Genetic Interactions | Lin-Smith-2010 |
| WDFY3 | GABRB3 | 0.00042331687 | Genetic Interactions | Lin-Smith-2010 |
| WDFY3 | GRIA1 | 0.0003349453 | Genetic Interactions | Lin-Smith-2010 |
| WDFY3 | CTNND2 | 0.00032242603 | Genetic Interactions | Lin-Smith-2010 |
| MED13 | GABRB3 | 0.0011483544 | Genetic Interactions | Lin-Smith-2010 |
| MED13 | GRIN2B | 0.0011284004 | Genetic Interactions | Lin-Smith-2010 |
| RELN | CNTN4 | 0.0002873026 | Genetic Interactions | Lin-Smith-2010 |
| RELN | KMT5B | 0.0005921976 | Genetic Interactions | Lin-Smith-2010 |
| RELN | CACNA1D | 0.0005954691 | Genetic Interactions | Lin-Smith-2010 |
| RELN | TBL1XR1 | 0.00031979682 | Genetic Interactions | Lin-Smith-2010 |
| RELN | ERBIN | 0.0005269484 | Genetic Interactions | Lin-Smith-2010 |
| RELN | RIMS1 | 0.00046273862 | Genetic Interactions | Lin-Smith-2010 |
| RELN | CTNND2 | 0.00031549265 | Genetic Interactions | Lin-Smith-2010 |
| RELN | GRIN2B | 0.00040701657 | Genetic Interactions | Lin-Smith-2010 |
| SMARCC2 | CTNND2 | 0.0007309388 | Genetic Interactions | Lin-Smith-2010 |
| SMARCC2 | GRIN2B | 0.0009429829 | Genetic Interactions | Lin-Smith-2010 |
| PHF3 | CACNA2D3 | 0.00027431492 | Genetic Interactions | Lin-Smith-2010 |
| PHF3 | KMT5B | 0.00049759547 | Genetic Interactions | Lin-Smith-2010 |
| PHF3 | INTS6 | 0.00051961414 | Genetic Interactions | Lin-Smith-2010 |
| PHF3 | ANK2 | 0.000309279 | Genetic Interactions | Lin-Smith-2010 |
| PHF3 | CACNA1D | 0.0005003443 | Genetic Interactions | Lin-Smith-2010 |
| PHF3 | TBL1XR1 | 0.00026871008 | Genetic Interactions | Lin-Smith-2010 |
| PHF3 | GIGYF2 | 0.0006220201 | Genetic Interactions | Lin-Smith-2010 |
| PHF3 | MED13L | 0.0004180429 | Genetic Interactions | Lin-Smith-2010 |
| PHF3 | NCKAP1 | 0.0005155805 | Genetic Interactions | Lin-Smith-2010 |
| PHF3 | GRIN2B | 0.00034199664 | Genetic Interactions | Lin-Smith-2010 |
| PHF3 | MED13 | 0.0010949785 | Genetic Interactions | Lin-Smith-2010 |
| SRSF11 | PTCHD1 | 0.0012463416 | Genetic Interactions | Lin-Smith-2010 |
| SRSF11 | GABRB3 | 0.0004411483 | Genetic Interactions | Lin-Smith-2010 |
| SRSF11 | MET | 0.0006875531 | Genetic Interactions | Lin-Smith-2010 |
| SRSF11 | ERBIN | 0.00056121335 | Genetic Interactions | Lin-Smith-2010 |
| SRSF11 | DSCAM | 0.00055146555 | Genetic Interactions | Lin-Smith-2010 |
| SRSF11 | TNRC6B | 0.0009064989 | Genetic Interactions | Lin-Smith-2010 |
| SRSF11 | CTNND2 | 0.00033600765 | Genetic Interactions | Lin-Smith-2010 |
| SRSF11 | PHF3 | 0.00042064363 | Genetic Interactions | Lin-Smith-2010 |
| CUL3 | ASH1L | 0.00079779705 | Genetic Interactions | Lin-Smith-2010 |
| CUL3 | TBL1XR1 | 0.00066432887 | Genetic Interactions | Lin-Smith-2010 |
| CUL3 | GABRB3 | 0.00086046604 | Genetic Interactions | Lin-Smith-2010 |
| CUL3 | GRIN2B | 0.00084551447 | Genetic Interactions | Lin-Smith-2010 |
| CUL3 | WDFY3 | 0.0009979171 | Genetic Interactions | Lin-Smith-2010 |
| CUL3 | PHF3 | 0.0008204713 | Genetic Interactions | Lin-Smith-2010 |
| UBN1 | TNRC6B | 0.0023612238 | Genetic Interactions | Lin-Smith-2010 |
| IRF2BP2 | CNTN4 | 0.00060212094 | Genetic Interactions | Lin-Smith-2010 |
| IRF2BP2 | SCN9A | 0.0017826882 | Genetic Interactions | Lin-Smith-2010 |
| IRF2BP2 | SRSF11 | 0.0010491769 | Genetic Interactions | Lin-Smith-2010 |
| NCKAP1L | NCKAP1 | 0.0015180805 | Genetic Interactions | Lin-Smith-2010 |
| NCKAP1L | MED13 | 0.003224066 | Genetic Interactions | Lin-Smith-2010 |
| NCKAP1L | PHF3 | 0.0009771528 | Genetic Interactions | Lin-Smith-2010 |
| MED12 | GRIA1 | 0.0022645348 | Genetic Interactions | Lin-Smith-2010 |
| MED12 | CTNND2 | 0.0021798934 | Genetic Interactions | Lin-Smith-2010 |
| GIGYF1 | WDFY3 | 0.0019501321 | Genetic Interactions | Lin-Smith-2010 |
| GIGYF1 | PHF3 | 0.001603367 | Genetic Interactions | Lin-Smith-2010 |
| CD2BP2 | GRIA1 | 0.00054933195 | Genetic Interactions | Lin-Smith-2010 |
| CD2BP2 | DSCAM | 0.0008678814 | Genetic Interactions | Lin-Smith-2010 |
| CD2BP2 | RIMS1 | 0.0007755997 | Genetic Interactions | Lin-Smith-2010 |
| CD2BP2 | GRIN2B | 0.00068220345 | Genetic Interactions | Lin-Smith-2010 |
| ST18 | CACNA2D3 | 0.00037203476 | Genetic Interactions | Lin-Smith-2010 |
| ST18 | KMT5B | 0.000674855 | Genetic Interactions | Lin-Smith-2010 |
| ST18 | ANK2 | 0.00041945418 | Genetic Interactions | Lin-Smith-2010 |
| ST18 | SCN9A | 0.0009693376 | Genetic Interactions | Lin-Smith-2010 |
| ST18 | NAA15 | 0.00070128095 | Genetic Interactions | Lin-Smith-2010 |
| ST18 | ERBIN | 0.0006004985 | Genetic Interactions | Lin-Smith-2010 |
| ST18 | KDM6A | 0.0028515153 | Genetic Interactions | Lin-Smith-2010 |
| ST18 | CTNND2 | 0.00035952835 | Genetic Interactions | Lin-Smith-2010 |
| ST18 | WDFY3 | 0.000547431 | Genetic Interactions | Lin-Smith-2010 |
| ST18 | UBN1 | 0.001485998 | Genetic Interactions | Lin-Smith-2010 |
| MYT1 | ANK2 | 0.0022698946 | Genetic Interactions | Lin-Smith-2010 |
| SCN4B | NAA15 | 0.0009407708 | Genetic Interactions | Lin-Smith-2010 |
| SCN4B | KDM6A | 0.0038253176 | Genetic Interactions | Lin-Smith-2010 |
| SCN4B | PHF3 | 0.00060379575 | Genetic Interactions | Lin-Smith-2010 |
| TNRC6A | GABRB3 | 0.0017091236 | Genetic Interactions | Lin-Smith-2010 |
| TNRC6A | KAT2B | 0.0036228325 | Genetic Interactions | Lin-Smith-2010 |
| NRXN3 | CNTN4 | 0.00018813719 | Genetic Interactions | Lin-Smith-2010 |
| NRXN3 | CACNA2D3 | 0.00021378377 | Genetic Interactions | Lin-Smith-2010 |
| NRXN3 | RANBP17 | 0.00049940316 | Genetic Interactions | Lin-Smith-2010 |
| NRXN3 | KMT5B | 0.00038779457 | Genetic Interactions | Lin-Smith-2010 |
| NRXN3 | DIP2C | 0.00035894758 | Genetic Interactions | Lin-Smith-2010 |
| NRXN3 | ANK2 | 0.0002410326 | Genetic Interactions | Lin-Smith-2010 |
| NRXN3 | CACNA1D | 0.00038993687 | Genetic Interactions | Lin-Smith-2010 |
| NRXN3 | ASH1L | 0.00025148873 | Genetic Interactions | Lin-Smith-2010 |
| NRXN3 | TBL1XR1 | 0.00020941571 | Genetic Interactions | Lin-Smith-2010 |
| NRXN3 | GABRB3 | 0.00027124383 | Genetic Interactions | Lin-Smith-2010 |
| NRXN3 | ERBIN | 0.00034506683 | Genetic Interactions | Lin-Smith-2010 |
| NRXN3 | TNRC6B | 0.0005573686 | Genetic Interactions | Lin-Smith-2010 |
| NRXN3 | SCN2A | 0.00034751583 | Genetic Interactions | Lin-Smith-2010 |
| NRXN3 | GRIN2B | 0.00026653067 | Genetic Interactions | Lin-Smith-2010 |
| NRXN3 | CD2BP2 | 0.0005159191 | Genetic Interactions | Lin-Smith-2010 |
| GRIA4 | CACNA1D | 0.0005000975 | Genetic Interactions | Lin-Smith-2010 |
| GRIA4 | GABRB3 | 0.00034787258 | Genetic Interactions | Lin-Smith-2010 |
| GRIA4 | DSCAM | 0.00043486455 | Genetic Interactions | Lin-Smith-2010 |
| GRIA4 | CD2BP2 | 0.00066167087 | Genetic Interactions | Lin-Smith-2010 |
| MED28 | CACNA2D3 | 0.00037124776 | Genetic Interactions | Lin-Smith-2010 |
| MED28 | IRF2BPL | 0.0009960771 | Genetic Interactions | Lin-Smith-2010 |
| MED28 | ANK2 | 0.0004185669 | Genetic Interactions | Lin-Smith-2010 |
| MED28 | CACNA1D | 0.0006771477 | Genetic Interactions | Lin-Smith-2010 |
| MED28 | ASH1L | 0.00043672457 | Genetic Interactions | Lin-Smith-2010 |
| MED28 | TBL1XR1 | 0.00036366237 | Genetic Interactions | Lin-Smith-2010 |
| MED28 | ERBIN | 0.00059922825 | Genetic Interactions | Lin-Smith-2010 |
| MED28 | RIMS1 | 0.00052621105 | Genetic Interactions | Lin-Smith-2010 |
| MED28 | WDFY3 | 0.00054627296 | Genetic Interactions | Lin-Smith-2010 |
| MED28 | RELN | 0.000534526 | Genetic Interactions | Lin-Smith-2010 |
| IRF2BP1 | NLGN3 | 0.011535212 | Genetic Interactions | Lin-Smith-2010 |
| IRF2BP1 | TBL1XR1 | 0.0013258123 | Genetic Interactions | Lin-Smith-2010 |
| IRF2BP1 | MED13L | 0.002062619 | Genetic Interactions | Lin-Smith-2010 |
| IRF2BP1 | ST18 | 0.0022207333 | Genetic Interactions | Lin-Smith-2010 |
| GRIA2 | KATNAL2 | 0.00059764116 | Genetic Interactions | Lin-Smith-2010 |
| GRIA2 | CNTN4 | 0.0002532221 | Genetic Interactions | Lin-Smith-2010 |
| GRIA2 | RANBP17 | 0.0006721686 | Genetic Interactions | Lin-Smith-2010 |
| GRIA2 | TCF20 | 0.00077234826 | Genetic Interactions | Lin-Smith-2010 |
| GRIA2 | DSCAM | 0.00045637367 | Genetic Interactions | Lin-Smith-2010 |
| GRIA2 | RIMS1 | 0.00040784752 | Genetic Interactions | Lin-Smith-2010 |
| GRIA2 | CTNND2 | 0.0002780682 | Genetic Interactions | Lin-Smith-2010 |
| GRIA2 | SCN2A | 0.00046773683 | Genetic Interactions | Lin-Smith-2010 |
| GRIA2 | WDFY3 | 0.00042339682 | Genetic Interactions | Lin-Smith-2010 |
| GRIA2 | USP7 | 0.0016949117 | Genetic Interactions | Lin-Smith-2010 |
| GRIA2 | SRSF11 | 0.00044123162 | Genetic Interactions | Lin-Smith-2010 |
| GRIA2 | NCKAP1L | 0.0010249786 | Genetic Interactions | Lin-Smith-2010 |
| GRIA2 | ST18 | 0.00047211803 | Genetic Interactions | Lin-Smith-2010 |
| GRIA2 | NRXN3 | 0.00027129502 | Genetic Interactions | Lin-Smith-2010 |
| DST | CACNA2D3 | 0.00039832527 | Genetic Interactions | Lin-Smith-2010 |
| DST | KMT2C | 0.0006780119 | Genetic Interactions | Lin-Smith-2010 |
| DST | CACNA1D | 0.0007265365 | Genetic Interactions | Lin-Smith-2010 |
| DST | GRIA1 | 0.00039988145 | Genetic Interactions | Lin-Smith-2010 |
| DST | SPAST | 0.0019425948 | Genetic Interactions | Lin-Smith-2010 |
| DST | DSCAM | 0.00063176674 | Genetic Interactions | Lin-Smith-2010 |
| DST | WDFY3 | 0.0005861162 | Genetic Interactions | Lin-Smith-2010 |
| DST | CD2BP2 | 0.00096126855 | Genetic Interactions | Lin-Smith-2010 |
| DST | MED28 | 0.0006521794 | Genetic Interactions | Lin-Smith-2010 |
| GRIN2B | GRIA1 | 0.07991372 | Pathway | Wu-Stein-2010 |
| RELN | GRIN2B | 0.033368457 | Pathway | Wu-Stein-2010 |
| KAT2B | GRIP1 | 0.032901216 | Pathway | Wu-Stein-2010 |
| KAT2B | SMARCC2 | 0.015032031 | Pathway | Wu-Stein-2010 |
| MED12 | MED13 | 0.048993323 | Pathway | Wu-Stein-2010 |
| CD2BP2 | SRSF11 | 0.008483524 | Pathway | Wu-Stein-2010 |
| GRIA2 | GRIN2B | 0.07991372 | Pathway | Wu-Stein-2010 |
| DST | ERBIN | 0.7409259 | Pathway | Wu-Stein-2010 |
| RELN | GRIN2B | 0.11027221 | Pathway | NCI_NATURE |
| MED13 | MED13L | 0.35380355 | Physical Interactions | Sato-Conaway-2004 |
| MED12 | MED13 | 0.2625878 | Physical Interactions | Sato-Conaway-2004 |
| MED28 | MED13L | 0.072282776 | Physical Interactions | Sato-Conaway-2004 |
| MED28 | MED13 | 0.06637544 | Physical Interactions | Sato-Conaway-2004 |
| MED28 | MED12 | 0.053647216 | Physical Interactions | Sato-Conaway-2004 |
| MED9 | MED13L | 0.06764718 | Physical Interactions | Sato-Conaway-2004 |
| MED9 | MED13 | 0.06211869 | Physical Interactions | Sato-Conaway-2004 |
| MED9 | MED12 | 0.050206747 | Physical Interactions | Sato-Conaway-2004 |
| MED9 | MED28 | 0.012690974 | Physical Interactions | Sato-Conaway-2004 |
| NRXN1 | NLGN3 | 0.060627203 | Physical Interactions | IREF-HPRD |
| CTNND2 | ERBIN | 0.06938213 | Physical Interactions | IREF-HPRD |
| MED13 | MED13L | 0.7187659 | Physical Interactions | IREF-HPRD |
| KAT2B | SRCAP | 0.053377558 | Physical Interactions | IREF-HPRD |
| MED12 | MED13 | 0.20798768 | Physical Interactions | IREF-HPRD |
| SCN4B | SCN2A | 0.61247575 | Physical Interactions | IREF-HPRD |
| NRXN3 | NLGN3 | 0.43694887 | Physical Interactions | IREF-HPRD |
| GRIA4 | GRIA1 | 0.081663676 | Physical Interactions | IREF-HPRD |
| NRXN2 | NLGN3 | 0.26575148 | Physical Interactions | IREF-HPRD |
| MED9 | MED13 | 0.07108297 | Physical Interactions | IREF-HPRD |
| MED9 | MED12 | 0.057234544 | Physical Interactions | IREF-HPRD |
| MED9 | MED28 | 0.063696355 | Physical Interactions | IREF-HPRD |
| DST | ERBIN | 0.0839854 | Physical Interactions | IREF-HPRD |
| CUL3 | TNRC6B | 0.0066835457 | Physical Interactions | Bennett-Harper-2010 |
| CUL3 | SMARCC2 | 0.036912974 | Physical Interactions | Bennett-Harper-2010 |
| CUL3 | USP7 | 0.009969304 | Physical Interactions | Bennett-Harper-2010 |
| CUL3 | SRSF11 | 0.020393869 | Physical Interactions | Bennett-Harper-2010 |
| CUL3 | ILF2 | 0.005439328 | Physical Interactions | Bennett-Harper-2010 |
| CD2BP2 | CUL3 | 0.036912974 | Physical Interactions | Bennett-Harper-2010 |
| TNRC6A | CUL3 | 0.036912974 | Physical Interactions | Bennett-Harper-2010 |
| GRIA1 | GRIP1 | 0.032829814 | Physical Interactions | IREF-BIOGRID |
| NRXN1 | NLGN3 | 0.133067 | Physical Interactions | IREF-BIOGRID |
| KDM6A | KMT2C | 0.06473425 | Physical Interactions | IREF-BIOGRID |
| TNRC6B | GIGYF2 | 0.037264876 | Physical Interactions | IREF-BIOGRID |
| GRIN2B | ERBIN | 0.019168315 | Physical Interactions | IREF-BIOGRID |
| KDM5B | CACNA1H | 0.07918546 | Physical Interactions | IREF-BIOGRID |
| KDM5B | GRIN2B | 0.018463274 | Physical Interactions | IREF-BIOGRID |
| SMARCC2 | KDM5B | 0.008374308 | Physical Interactions | IREF-BIOGRID |
| CUL3 | ERBIN | 0.0010379273 | Physical Interactions | IREF-BIOGRID |
| CUL3 | TNRC6B | 0.0025276334 | Physical Interactions | IREF-BIOGRID |
| CUL3 | SMARCC2 | 0.0010743726 | Physical Interactions | IREF-BIOGRID |
| CUL3 | USP7 | 0.00052711624 | Physical Interactions | IREF-BIOGRID |
| CUL3 | SRSF11 | 0.0013946685 | Physical Interactions | IREF-BIOGRID |
| CUL3 | ILF2 | 0.00061761285 | Physical Interactions | IREF-BIOGRID |
| MED12 | MED13 | 0.025457013 | Physical Interactions | IREF-BIOGRID |
| CD2BP2 | TBL1XR1 | 0.02861939 | Physical Interactions | IREF-BIOGRID |
| CD2BP2 | CUL3 | 0.0013959476 | Physical Interactions | IREF-BIOGRID |
| SCN4B | SCN2A | 0.8591811 | Physical Interactions | IREF-BIOGRID |
| TNRC6A | CUL3 | 0.0032936803 | Physical Interactions | IREF-BIOGRID |
| NRXN3 | NLGN3 | 0.29926524 | Physical Interactions | IREF-BIOGRID |
| GRIA4 | GRIP1 | 0.08600648 | Physical Interactions | IREF-BIOGRID |
| GRIA4 | GRIA1 | 0.13587937 | Physical Interactions | IREF-BIOGRID |
| MED28 | MED13L | 0.05109948 | Physical Interactions | IREF-BIOGRID |
| MED28 | MED13 | 0.021372842 | Physical Interactions | IREF-BIOGRID |
| MED28 | MED12 | 0.01319245 | Physical Interactions | IREF-BIOGRID |
| GRIA2 | GRIP1 | 0.042030547 | Physical Interactions | IREF-BIOGRID |
| GRIA2 | GRIA1 | 0.06640295 | Physical Interactions | IREF-BIOGRID |
| MED9 | MED13L | 0.07661285 | Physical Interactions | IREF-BIOGRID |
| MED9 | MED13 | 0.032044053 | Physical Interactions | IREF-BIOGRID |
| MED9 | MED12 | 0.019779285 | Physical Interactions | IREF-BIOGRID |
| MED9 | MED28 | 0.016606016 | Physical Interactions | IREF-BIOGRID |
| DST | ERBIN | 0.016690573 | Physical Interactions | IREF-BIOGRID |
| NRXN1 | NLGN3 | 0.21576788 | Predicted | I2D-BIND-Rat2Human |
| NRXN3 | NLGN3 | 0.3288982 | Predicted | I2D-BIND-Rat2Human |
| GRIA4 | GRIA1 | 0.53899354 | Predicted | I2D-BIND-Rat2Human |
| GRIA2 | GRIP1 | 0.06298361 | Predicted | I2D-BIND-Rat2Human |
| GRIA2 | GRIA1 | 0.10898389 | Predicted | I2D-BIND-Rat2Human |
| NRXN2 | NLGN3 | 0.3288982 | Predicted | I2D-BIND-Rat2Human |
| GRIN2B | SHANK2 | 0.047897182 | Predicted | I2D-IntAct-Mouse2Human |
| GRIA2 | GRIA1 | 0.20638463 | Predicted | I2D-IntAct-Mouse2Human |
| GRIA2 | GRIA4 | 0.39718446 | Predicted | I2D-IntAct-Mouse2Human |
| GRIA1 | GRIP1 | 0.07568352 | Predicted | I2D-MINT-Rat2Human |
| GRIA4 | GRIP1 | 0.09678016 | Predicted | I2D-MINT-Rat2Human |
| GRIA2 | GRIP1 | 0.048484635 | Predicted | I2D-MINT-Rat2Human |
| NRXN1 | NLGN3 | 0.25279924 | Predicted | Wu-Stein-2010 |
| CTNND2 | ERBIN | 0.056868482 | Predicted | Wu-Stein-2010 |
| MED13 | MED13L | 0.038620677 | Predicted | Wu-Stein-2010 |
| KAT2B | SRCAP | 0.033099797 | Predicted | Wu-Stein-2010 |
| MED12 | MED13L | 0.05479872 | Predicted | Wu-Stein-2010 |
| SCN4B | SCN2A | 1 | Predicted | Wu-Stein-2010 |
| NRXN3 | NLGN3 | 0.27595463 | Predicted | Wu-Stein-2010 |
| GRIA4 | GRIA1 | 0.11476122 | Predicted | Wu-Stein-2010 |
| MED28 | MED13L | 0.02511694 | Predicted | Wu-Stein-2010 |
| MED28 | MED13 | 0.03558315 | Predicted | Wu-Stein-2010 |
| MED28 | MED12 | 0.05048878 | Predicted | Wu-Stein-2010 |
| GRIA2 | GRIA1 | 0.05536067 | Predicted | Wu-Stein-2010 |
| GRIA2 | GRIA4 | 0.11641639 | Predicted | Wu-Stein-2010 |
| NRXN2 | NLGN3 | 0.3738508 | Predicted | Wu-Stein-2010 |
| PHF3 | KDM5B | 0.04967078 | Predicted | Stuart-Kim-2003 |
| KMT2C | KMT5B | 0.008730745 | Shared protein domains | INTERPRO |
| INTS6 | CACNA2D3 | 0.010047774 | Shared protein domains | INTERPRO |
| SCN9A | CACNA1D | 0.007215458 | Shared protein domains | INTERPRO |
| ASH1L | KMT5B | 0.007480644 | Shared protein domains | INTERPRO |
| ASH1L | SRCAP | 0.007391455 | Shared protein domains | INTERPRO |
| ASH1L | KMT2C | 0.012824674 | Shared protein domains | INTERPRO |
| CACNA1H | CACNA1D | 0.01297956 | Shared protein domains | INTERPRO |
| CACNA1H | SCN9A | 0.011234983 | Shared protein domains | INTERPRO |
| SPAST | KATNAL2 | 0.011995117 | Shared protein domains | INTERPRO |
| DSCAM | CNTN4 | 0.014710028 | Shared protein domains | INTERPRO |
| SCN2A | CACNA1D | 0.00678411 | Shared protein domains | INTERPRO |
| SCN2A | SCN9A | 0.05176127 | Shared protein domains | INTERPRO |
| SCN2A | CACNA1H | 0.010563344 | Shared protein domains | INTERPRO |
| GRIN2B | GRIA1 | 0.041564234 | Shared protein domains | INTERPRO |
| KDM5B | TCF20 | 0.009323023 | Shared protein domains | INTERPRO |
| WDFY3 | RIMS1 | 0.010208495 | Shared protein domains | INTERPRO |
| MED13 | MED13L | 1 | Shared protein domains | INTERPRO |
| PHF3 | KMT2C | 0.0074760797 | Shared protein domains | INTERPRO |
| PHF3 | TCF20 | 0.010670796 | Shared protein domains | INTERPRO |
| PHF3 | ASH1L | 0.008845667 | Shared protein domains | INTERPRO |
| PHF3 | KDM5B | 0.009223224 | Shared protein domains | INTERPRO |
| SRSF11 | TNRC6B | 0.004351803 | Shared protein domains | INTERPRO |
| UBN1 | UBN2 | 1 | Shared protein domains | INTERPRO |
| IRF2BP2 | IRF2BPL | 0.7449552 | Shared protein domains | INTERPRO |
| NCKAP1L | NCKAP1 | 1 | Shared protein domains | INTERPRO |
| GIGYF1 | GIGYF2 | 0.42737535 | Shared protein domains | INTERPRO |
| CD2BP2 | GIGYF2 | 0.42737535 | Shared protein domains | INTERPRO |
| CD2BP2 | GIGYF1 | 0.42737535 | Shared protein domains | INTERPRO |
| ST18 | MYT1L | 0.31383395 | Shared protein domains | INTERPRO |
| MYT1 | MYT1L | 0.31383395 | Shared protein domains | INTERPRO |
| MYT1 | ST18 | 0.31383395 | Shared protein domains | INTERPRO |
| TNRC6A | TNRC6B | 0.10694546 | Shared protein domains | INTERPRO |
| TNRC6A | SRSF11 | 0.0041781254 | Shared protein domains | INTERPRO |
| NRXN3 | NRXN1 | 0.039292276 | Shared protein domains | INTERPRO |
| GRIA4 | GRIA1 | 0.041972898 | Shared protein domains | INTERPRO |
| GRIA4 | GRIN2B | 0.04106463 | Shared protein domains | INTERPRO |
| RP11-6L6.2 | RELN | 0.1479676 | Shared protein domains | INTERPRO |
| IRF2BP1 | IRF2BPL | 0.22989899 | Shared protein domains | INTERPRO |
| IRF2BP1 | IRF2BP2 | 0.22989899 | Shared protein domains | INTERPRO |
| GRIA2 | GRIA1 | 0.041972898 | Shared protein domains | INTERPRO |
| GRIA2 | GRIN2B | 0.04106463 | Shared protein domains | INTERPRO |
| GRIA2 | GRIA4 | 0.05723623 | Shared protein domains | INTERPRO |
| NRXN2 | NRXN1 | 0.03696346 | Shared protein domains | INTERPRO |
| NRXN2 | NRXN3 | 0.03696346 | Shared protein domains | INTERPRO |
| RP5-1022E24.6 | MYT1L | 0.15692404 | Shared protein domains | INTERPRO |
| RP5-1022E24.6 | ST18 | 0.15692404 | Shared protein domains | INTERPRO |
| RP5-1022E24.6 | MYT1 | 0.15692404 | Shared protein domains | INTERPRO |
| KMT2C | KMT5B | 0.018382039 | Shared protein domains | PFAM |
| INTS6 | CACNA2D3 | 0.012840409 | Shared protein domains | PFAM |
| SCN9A | CACNA1D | 0.010026993 | Shared protein domains | PFAM |
| ASH1L | KMT5B | 0.01497782 | Shared protein domains | PFAM |
| ASH1L | KMT2C | 0.015233556 | Shared protein domains | PFAM |
| TBL1XR1 | KATNAL2 | 0.02496287 | Shared protein domains | PFAM |
| CACNA1H | CACNA1D | 0.012148273 | Shared protein domains | PFAM |
| CACNA1H | SCN9A | 0.01230882 | Shared protein domains | PFAM |
| ERBIN | GRIP1 | 0.016491229 | Shared protein domains | PFAM |
| SPAST | KATNAL2 | 0.015843533 | Shared protein domains | PFAM |
| DSCAM | CNTN4 | 0.016319748 | Shared protein domains | PFAM |
| SCN2A | CACNA1D | 0.009703854 | Shared protein domains | PFAM |
| SCN2A | SCN9A | 0.055226352 | Shared protein domains | PFAM |
| SCN2A | CACNA1H | 0.011912147 | Shared protein domains | PFAM |
| GRIN2B | GRIA1 | 0.04271426 | Shared protein domains | PFAM |
| KDM5B | KDM6A | 0.00989325 | Shared protein domains | PFAM |
| MED13 | MED13L | 1 | Shared protein domains | PFAM |
| PHF3 | KMT2C | 0.011419423 | Shared protein domains | PFAM |
| PHF3 | KDM5B | 0.009068125 | Shared protein domains | PFAM |
| UBN1 | UBN2 | 1 | Shared protein domains | PFAM |
| IRF2BP2 | IRF2BPL | 0.74584025 | Shared protein domains | PFAM |
| NCKAP1L | NCKAP1 | 1 | Shared protein domains | PFAM |
| GIGYF1 | GIGYF2 | 0.5 | Shared protein domains | PFAM |
| CD2BP2 | GIGYF2 | 0.5 | Shared protein domains | PFAM |
| CD2BP2 | GIGYF1 | 0.5 | Shared protein domains | PFAM |
| ST18 | MYT1L | 0.25465763 | Shared protein domains | PFAM |
| MYT1 | MYT1L | 0.25465763 | Shared protein domains | PFAM |
| MYT1 | ST18 | 0.25465763 | Shared protein domains | PFAM |
| TNRC6A | TNRC6B | 0.7927326 | Shared protein domains | PFAM |
| NRXN3 | NRXN1 | 0.03227324 | Shared protein domains | PFAM |
| GRIA4 | GRIA1 | 0.043422285 | Shared protein domains | PFAM |
| GRIA4 | GRIN2B | 0.041839138 | Shared protein domains | PFAM |
| RP11-6L6.2 | RELN | 0.49305186 | Shared protein domains | PFAM |
| IRF2BP1 | IRF2BPL | 0.19752897 | Shared protein domains | PFAM |
| IRF2BP1 | IRF2BP2 | 0.19752897 | Shared protein domains | PFAM |
| GRIA2 | GRIA1 | 0.043422285 | Shared protein domains | PFAM |
| GRIA2 | GRIN2B | 0.041839138 | Shared protein domains | PFAM |
| GRIA2 | GRIA4 | 0.06910934 | Shared protein domains | PFAM |
| NRXN2 | NRXN1 | 0.039018974 | Shared protein domains | PFAM |
| NRXN2 | NRXN3 | 0.039166182 | Shared protein domains | PFAM |
| RP5-1022E24.6 | MYT1L | 0.25465763 | Shared protein domains | PFAM |
| RP5-1022E24.6 | ST18 | 0.25465763 | Shared protein domains | PFAM |
| RP5-1022E24.6 | MYT1 | 0.25465763 | Shared protein domains | PFAM |

**Supplementary Table 3.** Biological functions and FDR for those genes reported by GeneMANIA to construct the network of SFARI genes with scores 1 and 2 carrying DNMs.

| Function | FDR | Genes in network | Genes in genome |
| --- | --- | --- | --- |
| neuron cell-cell adhesion | 0.000005738863954955389 | 5 | 13 |
| vocalization behavior | 0.000005738863954955389 | 5 | 11 |
| neuron part | 0.000005738863954955389 | 12 | 298 |
| glutamate receptor signaling pathway | 0.000005738863954955389 | 7 | 52 |
| cognition | 0.00003199056814608648 | 7 | 70 |
| social behavior | 0.00003818502367930477 | 5 | 23 |
| behavior | 0.00003818502367930477 | 10 | 228 |
| neuron projection | 0.00003818502367930477 | 10 | 233 |
| ion channel complex | 0.00003818502367930477 | 8 | 121 |
| ionotropic glutamate receptor complex | 0.00003818502367930477 | 5 | 22 |
| intraspecies interaction between organisms | 0.00003818502367930477 | 5 | 23 |
| transmembrane transporter complex | 0.00006562272635209062 | 8 | 134 |
| membrane depolarization | 0.00007122047167043318 | 6 | 53 |
| multi-organism behavior | 0.00009709901065942655 | 5 | 29 |
| learning or memory | 0.00009709901065942655 | 6 | 57 |
| learning | 0.00012948007997587167 | 5 | 31 |
| adult behavior | 0.00016922465756414944 | 5 | 33 |
| regulation of membrane potential | 0.00021937580846060863 | 8 | 165 |
| positive regulation of synaptic transmission | 0.00025621531002144126 | 4 | 15 |
| ionotropic glutamate receptor signaling pathway | 0.00032361174432913063 | 4 | 16 |
| regulation of ion transmembrane transporter activity | 0.0012192332491429438 | 6 | 92 |
| single-organism behavior | 0.0012403780843713854 | 6 | 93 |
| regulation of transmembrane transporter activity | 0.0012635752634726273 | 6 | 94 |
| axon | 0.0014565706566496833 | 6 | 97 |
| regulation of transporter activity | 0.0022237027333877547 | 6 | 105 |
| regulation of ion transmembrane transport | 0.004422246740213047 | 6 | 119 |
| gated channel activity | 0.005041307451159044 | 7 | 188 |
| regulation of synaptic transmission | 0.005041307451159044 | 5 | 71 |
| positive regulation of excitatory postsynaptic membrane potential | 0.005119735873232188 | 3 | 11 |
| membrane depolarization during cardiac muscle cell action potential | 0.005119735873232188 | 3 | 11 |
| ionotropic glutamate receptor activity | 0.005119735873232188 | 3 | 11 |
| regulation of transmembrane transport | 0.005119735873232188 | 6 | 126 |
| postsynaptic membrane organization | 0.006224243931803185 | 3 | 12 |
| regulation of synaptic transmission, glutamatergic | 0.006224243931803185 | 3 | 12 |
| membrane assembly | 0.006224243931803185 | 3 | 12 |
| membrane depolarization during action potential | 0.007633343800975214 | 3 | 13 |
| alpha-amino-3-hydroxy-5-methyl-4-isoxazolepropionic acid selective glutamate receptor complex | 0.007633343800975214 | 3 | 13 |
| synapse organization | 0.007997193811028496 | 5 | 83 |
| synaptic transmission, glutamatergic | 0.008962117241929558 | 3 | 14 |
| positive regulation of membrane potential | 0.008962117241929558 | 3 | 14 |
| basal plasma membrane | 0.010640210461373622 | 3 | 15 |
| membrane biogenesis | 0.010640210461373622 | 3 | 15 |
| regulation of excitatory postsynaptic membrane potential | 0.012756359078983408 | 3 | 16 |
| regulation of alpha-amino-3-hydroxy-5-methyl-4-isoxazole propionate selective glutamate receptor activity | 0.015096720750698634 | 3 | 17 |
| ion channel activity | 0.016751741809032304 | 7 | 246 |
| regulation of postsynaptic membrane potential | 0.016751741809032304 | 3 | 18 |
| cell adhesion molecule binding | 0.016751741809032304 | 4 | 51 |
| basal part of cell | 0.016751741809032304 | 3 | 18 |
| transcription coactivator activity | 0.01722071406759326 | 7 | 249 |
| substrate-specific channel activity | 0.018764954859924736 | 7 | 254 |
| regulation of membrane repolarization | 0.018764954859924736 | 3 | 19 |
| regulation of heart rate by cardiac conduction | 0.02045484332303922 | 3 | 20 |
| synapse assembly | 0.02045484332303922 | 4 | 55 |
| glutamate receptor activity | 0.02045484332303922 | 3 | 20 |
| membrane repolarization | 0.02336651350773279 | 3 | 21 |
| regulation of glutamate receptor signaling pathway | 0.02558690529795177 | 3 | 22 |
| channel activity | 0.02558690529795177 | 7 | 272 |
| passive transmembrane transporter activity | 0.02558690529795177 | 7 | 272 |
| neurotransmitter secretion | 0.02591942571624791 | 4 | 60 |
| positive regulation of ion transmembrane transporter activity | 0.05111194982222823 | 3 | 28 |
| action potential | 0.05111194982222823 | 4 | 72 |
| cell communication involved in cardiac conduction | 0.05572462385925814 | 3 | 29 |
| regulation of ion transport | 0.05587267527487197 | 6 | 219 |
| cardiac muscle cell action potential | 0.05914000884940174 | 3 | 30 |
| neurotransmitter transport | 0.05914000884940174 | 4 | 76 |
| basolateral plasma membrane | 0.06126066151142516 | 4 | 77 |
| extracellular ligand-gated ion channel activity | 0.06942917191479611 | 3 | 32 |
| cardiac conduction | 0.07395751878459662 | 3 | 33 |
| neuron-neuron synaptic transmission | 0.07395751878459662 | 3 | 33 |
| positive regulation of transporter activity | 0.07973935682068578 | 3 | 34 |
| synapse | 0.08095326742537862 | 5 | 154 |
| regulation of neurotransmitter levels | 0.0821284912350024 | 4 | 85 |
| regulation of synapse organization | 0.08340426669470688 | 3 | 35 |
| regulation of heart contraction | 0.09526893594997858 | 4 | 89 |
